# Parallel Single-Cell RNA-Seq and Genetic Recording Reveals Lineage Decisions in Developing Embryoid Bodies

**DOI:** 10.1016/j.celrep.2020.108222

**Published:** 2020-10-06

**Authors:** Ik Soo Kim, Jingyi Wu, Gilbert J. Rahme, Sofia Battaglia, Atray Dixit, Elizabeth Gaskell, Huidong Chen, Luca Pinello, Bradley E. Bernstein

**Affiliations:** 1 Department of Pathology and Center for Cancer Research, Massachusetts General Hospital and Harvard Medical School, Boston, MA 02114, USA; 2 Broad Institute of Harvard and MIT, Cambridge, MA 02142, USA; 3 These authors contributed equally; 4 Lead Contact

## Abstract

Early developmental specification can be modeled by differentiating embryonic stem cells (ESCs) to embryoid bodies (EBs), a heterogeneous mixture of three germ layers. Here, we combine single-cell transcriptomics and genetic recording to characterize EB differentiation. We map transcriptional states along a time course and model cell fate trajectories and branchpoints as cells progress to distinct germ layers. To validate this inferential model, we propose an innovative inducible genetic recording technique that leverages recombination to generate cell-specific, timestamp barcodes in a narrow temporal window. We validate trajectory architecture and key branchpoints, including early specification of a primordial germ cell (PGC)-like lineage from preimplantation epiblast-like cells. We further identify a temporally defined role of DNA methylation in this PGC-epiblast decision. Our study provides a high-resolution lineage map for an organoid model of embryogenesis, insights into epigenetic determinants of fate specification, and a strategy for lineage mapping of rapid differentiation processes.

## In Brief

Kim et al. present a temporally precise genetic recording system for lineage tracing and transcriptomics analysis of single cells. They generate a trajectory map and single-cell transcriptional atlas of developing embryoid bodies, an organoid model of pre-gastrulation embryogenesis. These data reveal transcriptional and epigenetic regulators of early cell fate decisions.

## Graphical Abstract

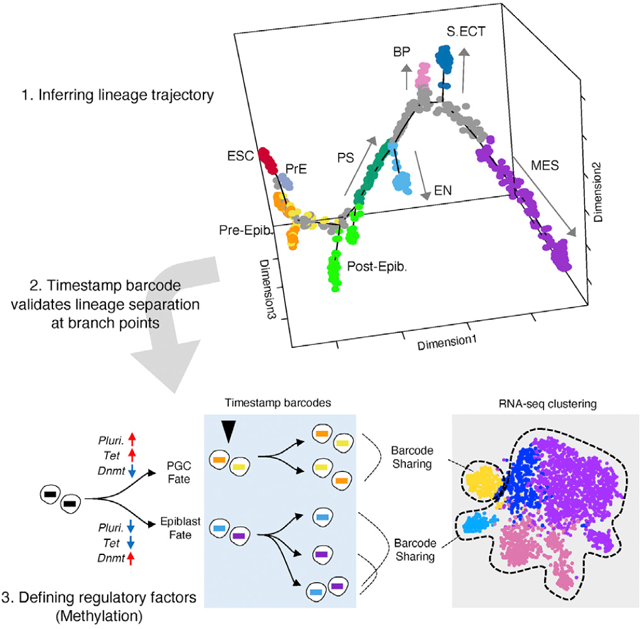

## INTRODUCTION

Development of a multicellular organism from a zygote is a complex process, tightly controlled by hierarchical transcriptional programs, epigenetic regulation, and spatial contexts. The process gives rise to all cell states through a sequence of precisely orchestrated cell divisions and specification events (Tam and Loebel, 2007). Classic studies of pre-gastrulation embryogenesis and *in vitro* models have led to a deep understanding of how lineage-specifying transcription factors and sequential epigenetic silencing of pluripotency genes contribute to each embryonic cell state (Keller, 2005; Takaoka and Hamada, 2012; Tam and Loebel, 2007; Theunissen and Jaenisch, 2017). However, our understanding of the cellular hierarchies and timing of specification events during this early time window has lagged, in part because of a lack of technologies for mapping lineage relationships with sufficient temporal resolution. Understanding these lineage relationships and the transcriptional and epigenetic programs that control them is critical for our understanding of the fundamental processes by which cell identity is established.

Single-cell transcriptomics and lineage trajectory modeling have enriched our understanding of cell states and their temporal relationships in the developing embryo (Boroviak et al., 2015; Bowling et al., 2020; Cao et al., 2019; Deng et al., 2014; Han et al., 2018; Lescroart et al., 2018; Mohammed et al., 2017; Ohnishi et al., 2014; Pijuan-Sala et al., 2019). When combined with genetic recorders, these technologies have the exciting potential to address long-standing questions in the field regarding lineage relationships (Kester and van Oudenaarden, 2018; McKenna and Gagnon, 2019). For example, CRISPR-Cas9-based genetic barcodes have been applied to map lineage relationships in later stages of mouse embryogenesis (later than embryonic day 8.5 [E8.5]), confirming intriguing relationships, such as the transcriptional convergence between extraembryonic and embryonic endoderm lineages (Chan et al., 2019; Nowotschin et al., 2019). However, current CRISPR-based barcoding technologies require many cell divisions to evolve sufficient complexity to infer lineage relationships, which limits their applicability to pre-gastrulation embryogenesis or other similarly rapid and complex developmental processes.

Here we use an *in vitro* system for differentiating mouse embryonic stem cells (ESCs) to embryoid bodies (EB) to map and perturb transcriptional programs that underlie cell fate specification. We map the developmental trajectories and cell states that unfold as the three germ layers form *in vitro*, and we relate them to *in vivo* cell states. To validate inferred trajectories, we develop a genetic recording system based on a rapid recombination event to generate cell-specific barcodes in narrow temporal windows during the time course. Implementation of this recording system validated key branchpoints in our EB time course, including early specification of a primordial germ cell (PGC)-like cell state from cells that closely resemble preimplantation epiblast-like cells. We show that the commitment of these PGC-like cells is directed by an early switch in the DNA methylation state, documenting the precise time window during which a critical epigenetic driver of early development operates.

## RESULTS

### Single-Cell Profiling and Reconstruction of the Developmental Trajectory

**Figure 1.**
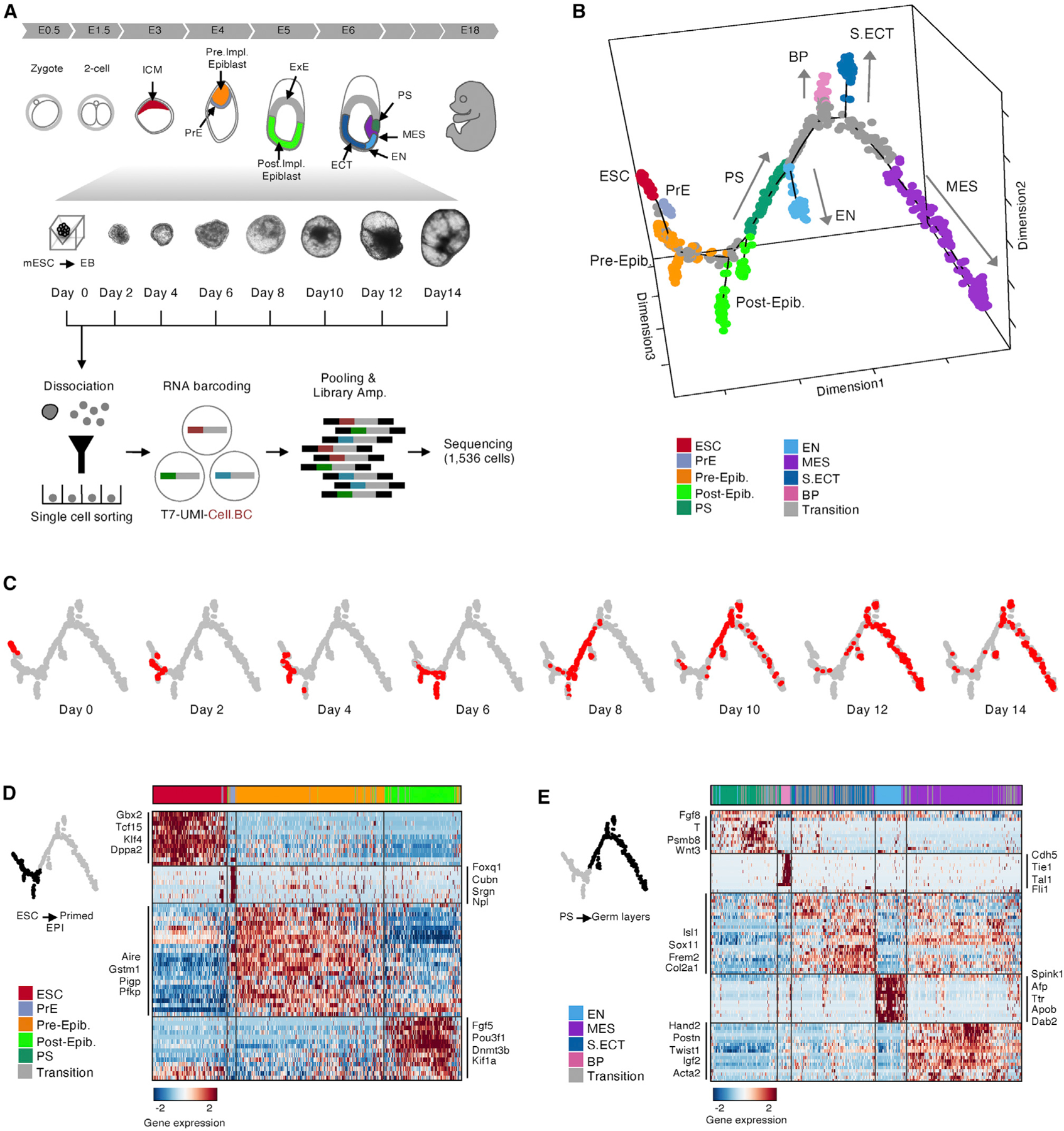
Single-Cell Profiling and Reconstruction of the Developmental Trajectory (A) Overview of the experimental design and the corresponding stages of embryogenesis. (B) Pseudotime trajectory of 1,536 single-cell transcriptomes (points) from all stages of the EB time course. Data are from two independent biological replicates. Trajectory was inferred by Monocle 2. Cells are color coded by cell-state annotations from the analyses in (D) and (E) and Figures S1D and S1F–S1H. Pre-Epib, preimplantation epiblast-like; Post-Epib, postimplantation epiblast-like; S. ECT, surface ectoderm (C) Pseudotime trajectory from (B), with cells from each real-time point superimposed in red. (D and E) Heatmaps showing unbiased clustering of meta-modules based on differentially expressed genes from the same 1,536 single-cell transcriptomes. Cells were split into two categories: early (ESC to epiblast; D) or late (PS to germ layers; E). The top differentially expressed genes for each cluster are annotated (p < 1e–5). Cells were assigned “transition” (gray) when they did not have a clear lineage identity. Cell states were assigned based on marker gene expression. See also Figure S1 and Table S3.

To generate EBs, we maintained mouse ESCs in medium supplemented with serum and leukemia inhibitory factor (LIF) and seeded ~1,000 cells per microwell into the same medium without LIF to initiate differentiation (Wilson et al., 2014). In the absence of LIF, the cultures aggregated spontaneously and differentiated into EBs over 14 days (Figure 1A; STAR Methods). ESCs in serum plus LIF correspond to day 0 in our time course. On day 1, cell aggregates begin to form, and by day 2, dense aggregates with visible substructures are present (Figure S1A). On day 14, EBs express markers of all three germ layers: mesoderm (MES), endoderm (EN), and ectoderm (ECT) (Figures S1B and S1C; STAR Methods; Keller, 2005; Murry and Keller, 2008; Tam and Loebel, 2007).

To characterize the transcriptome of the EBs at single-cell resolution, we isolated viable cells every 48 h for 14 days and performed deep transcriptomics profiling using CEL-seq2 (Hashimshony et al., 2016). This plate-based method has lower throughput than droplet-based single-cell technologies but obtains significantly higher numbers of transcripts per cell, enhancing the ability to distinguish cell states (Ding et al., 2019). We assigned RNA sequencing (RNA-seq) reads to individual cells based on their cell barcode, aligned them to the transcriptome, and counted individual mRNA molecules using unique molecular identifiers (UMIs) (STAR Methods). We acquired high-quality data from 1,536 cells that passed our quality control (QC) and gene complexity metrics, from two independent biological replicates. In total, approximately 80% of cells passed our QC (STAR Methods). For cells that passed QC, we detected a median of 44,197 UMIs for an average of 6,000 genes per cell.

Next we utilized the single-cell transcriptomics data to infer differentiation trajectories across the EB time course. We used Monocle 2, a graph-based machine learning approach that orders single-cell transcriptomes based on their similarity and puts out a graph of a “pseudotime course” (Figure 1B; Qiu et al., 2017). Monocle 2 assumes that the different trajectories can be described with a tree structure with different states, and each cell’s pseudotime value is the distance a cell would have to travel from a user-specified root state in this tree. This pseudotime course can therefore be considered a quantitative measure of progress through a biological process (in this case, differentiation) (Figure 1C). The reconstructed trajectory comprised 26 cell state clusters and 6 different terminal branches (Figure S1D; STAR Methods). We then merged these 26 clusters into 10 main cell populations by differential gene expression analysis and hierarchical clustering (Figures 1B, 1D, 1E, S1F, and S1G; STAR Methods). Increased pseudotime in the trajectory correlated with increased differentiation and decreased pluripotency, giving us confidence in the reconstruction (Figure S1E).

Cell populations expressed well known markers of early embryonic cell populations (Figures 1B, 1D, and 1E); for example, *Oct4*, *Nanog*, *Gbx2*, Klf4, and *Dppa2* for ESCs; *T* (*brachyury*), *Fgf8*, and *Wnt3* for primitive streak (PS)-like cells); and standard markers for the germ layers (Boroviak et al., 2015; Cao et al., 2019; Chan et al., 2019). We identified extraembryonic primitive EN (PrE)-like cells and EN-like cells based on PrE markers (*Foxq1*, *Cubn*, and *Srgn*) and EN markers (*Spink1*, *Afp*, and *Dab2*) (Figures 1D, 1E, and S1H; Gouon-Evans et al., 2006; Ohnishi et al., 2014). We also identified two epiblast-like cell clusters: preimplantation epiblast-like cells that arise early on days 2–4 and express *Aire*, *Pfkp*, and *Gstm1* and postimplantation epiblast-like cells that arise on day 6 and express *Fgf5*, *Pou3f1*, and *Dnmt3b* (Figures 1B and 1D; Boroviak et al., 2015). Finally, we annotated a small cluster of blood progenitor (BP)-like cells that distinctly express *Cdh5*, *Tie1*, *Tal1*, and *Fli1* (Gritz and Hirschi, 2016; Wang and Nakayama, 2009; Figures 1B and 1E). Our cluster annotations were also supported by independent Louvain clustering and tSNE analyses (Figure S1I). However, these algorithms, which do not incorporate pseudotime information, failed to distinguish PrE, EN, and BP and classified them with general EN (Figure S1I). All 10 cell states were identified in both independent biological replicates. In addition, all major lineages were present in each single EB analyzed (Figures S6A and S6B).

### Annotation of PGC-like Cells

**Figure 2.**
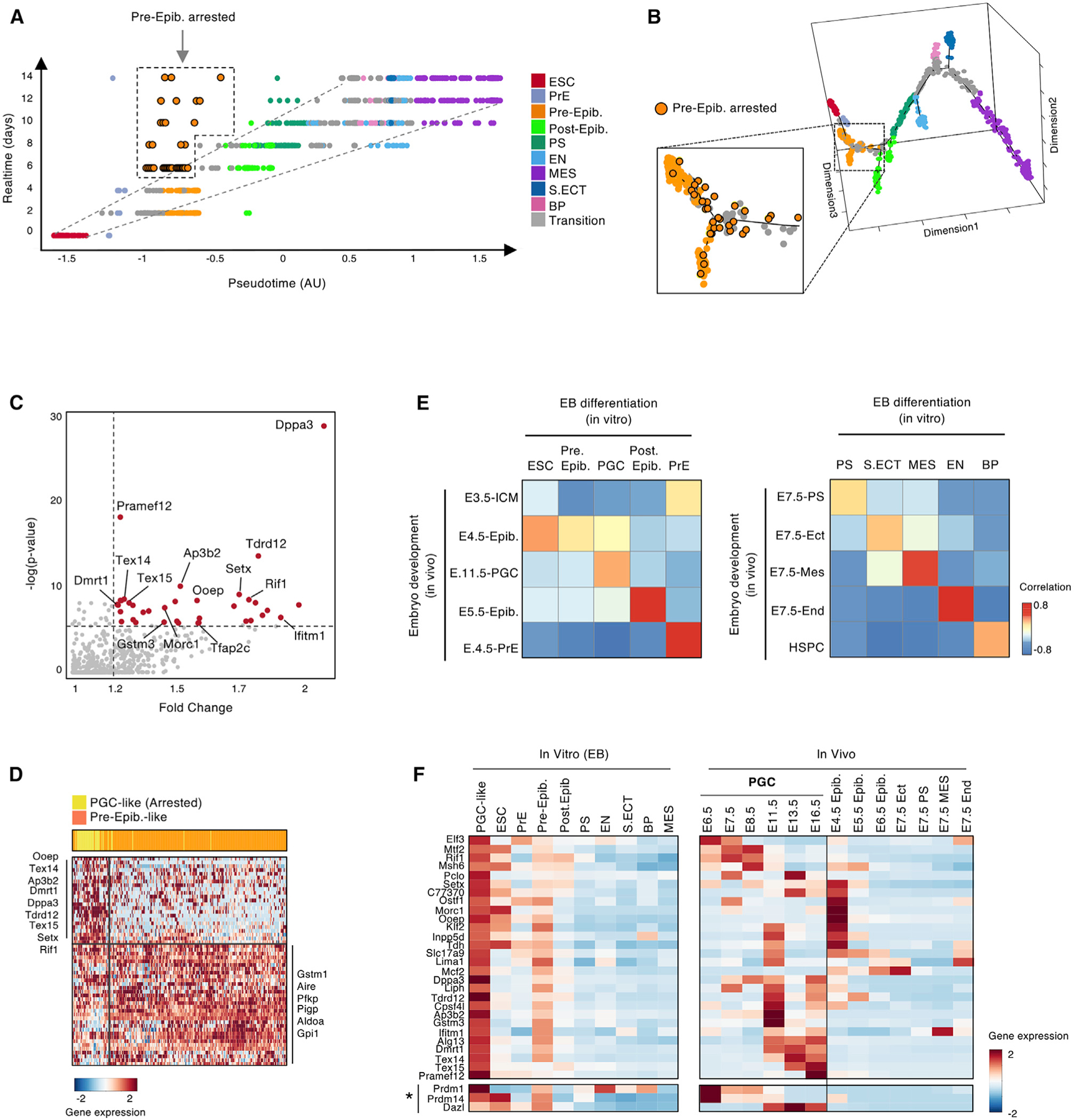
EB Differentiation Recapitulates the Developmental Trajectory of the Pre-gastrulation Embryo (A) Plot comparing real-time point of collection (y axis) with pseudotime score (x axis) for the same 1,536 single cells from Figure 1 (points). Cells are color coded by cell-state annotations from Figure 1. We identify a population of Pre-Epib cells with arrested differentiation (orange dots, black outline). (B) Pseudotime trajectory as in Figure 1B, with a magnified view of the cells with arrested differentiation (orange dots, black outline). These cells fall at the branchpoint of ESCs (red), Pre-Epib cells (orange), and Post-Epib cells (lime green). (C) Plot showing differentially expressed genes in the cell population with arrested differentiation. The top differentially expressed genes (36 genes, −log(p) > 5.8, fold change > 1.2) are highlighted (red). Top differentially expressed genes include many PGC marker genes. We therefore annotated the population of cells with arrested differentiation as PGC like. (D) Heatmap showing unbiased clustering of meta-modules based on the top 30 differentially expressed genes from the single-cell transcriptomes of cells annotated as PGC like (yellow) or Pre-Epib (orange). The top differentially expressed genes for each cluster are annotated (p < 1e–3). (E) Heatmap showing a correlation analysis of the annotated EB cell states with gene expression data from bulk RNA-seq from isolated populations *in vivo* (GEO: GSE76505; Zhang et al., 2018). (F) Heatmap (left) of genes that are preferentially expressed in the PGC-like population, showing their expression across EB cell states (*p > 1e–3). A heatmap (right) of the same genes shows their average expression across *in vivo* populations, taken from published RNA-seq data (GEO: GSE76505; E-MTAB-2958; Boroviak et al., 2015; Zhang et al., 2018). These analyses support annotation of the cells with arrested differentiation as PGC like. See also Figure S2 and Table S3.

Because the Monocle 2 graph orders single cells by similarity of their transcriptome, it assigns each cell a pseudotime score that reflects its divergence from the ESC state. The pseudotime score for individual cells correlated well with the actual time points of collection, with some notable exceptions (Figure 2A). In particular, we identified 36 preimplantation epiblast-like cells with a low pseudotime score despite having been detected at multiple time points of EB differentiation. This suggested that these cells were arrested in their differentiation (Figure 2A; STAR Methods).

These cells map to the trajectory at the branchpoint between ESCs, preimplantation epiblast-like cells, and postimplantation epiblast-like cells (Figures 2B and S2A). The most significant differentially expressed genes in this cluster are previously reported markers of PGCs, such as *Dppa3/stella*, *Ifitm1*, and *Tfap2c* (and its ortholog *Ap3b2*) (Chen et al., 2018; Tanaka et al., 2005), or of differentiated germ cells, such as *Tex14/15*, *Tdrd12*, and *Ooep* (Pandey et al., 2013; Pierre et al., 2007; Wang et al., 2001; Figures 2C; S2C–S2E). Unbiased clustering separated these 36 cells from the remainder of the preimplantation epiblast-like cells, supporting the conclusion that they represent a distinct population. We therefore annotated this cluster as PGC-like cells (Figure 2D).

### EB Differentiation Recapitulates the Developmental Trajectory of the Pre-gastrulation Embryo

We next compared the transcriptomes of the main populations identified in the EB differentiation course with the pre-gastrulation mouse embryo. We correlated the aggregated RNA profiles of our assigned cell lineages with annotated *in vivo* populations from prior bulk and single-cell studies (Figure 2E; Boroviak et al., 2015; Nestorowa et al., 2016; Seisenberger et al., 2012; Zhang et al., 2018; STAR Methods). Overall, the expression profiles of the major clusters from the EBs correlated well (R > 0.5) with annotated *in vivo* cell types. In addition, comparison with single-cell data for *in vivo* embryos (Argelaguet et al., 2019) suggested that the population we term EN is a mixture of definitive EN (DE) and extraembryonic visceral EN (VE) (Figures S2F–S2H). The presence of ESCs, epiblast-like cells, PSs, and the three germ layers, together with concordance with published *in vivo* datasets, suggests that our EB time course approximately recapitulates major cell types in stages E3.5–E7.5 of mouse embryogenesis, corresponding to the preimplantation blastocyst through early germ layer differentiation (Figures 1A, S2I, and S2J).

We also sought to determine how closely the PGC-like population resembled PGCs *in vivo*. PGCs are a specific unipotent cell state that arises *in vivo* from primed, postimplantation epiblast cells at ~E6.5 in response to BMP4 signaling from extraembryonic tissues (Ohinata et al., 2005, 2009; Yamaji et al., 2008). Formation of PGCs in EBs and other *in vitro* models is enhanced by addition of BMP4 and other extrinsic signaling factors (Irie et al., 2014; Keller, 2005; Magnúsdóttir et al., 2013; Nakaki et al., 2013). Extrinsic BMP4 in our system is provided by serum. Although genes immediately downstream of BMP4 signaling, such as *Blimp1/Prdm1* and *Prdm14*, are expressed in our *in vitro* PGC-like cells, they do not reach statistical significance in a differential gene expression analysis. Next we compared EB-derived PGC-like cells with isolated embryonic tissues at various stages (Boroviak et al., 2015; Magnúsdóttir et al., 2013; Seisenberger et al., 2012; Zhang et al., 2018), which suggested that EB-derived PGC-like cells share transcriptional programs with E4.5 preimplantation epiblast cells and PGCs isolated from E11.5 embryos (Figure 2F). PGC-like cells also appeared to be cycling slowly (Figure S2B), but we found no evidence that they progress to mature germ cells during the EB time course, potentially because other extrinsic cues are lacking in this organoid system (Figure S6I).

Taken together, the real-time and pseudotime information reveal that the major lineages arise spontaneously in EBs in the same strict temporal order that occurs in the developing embryo (Figures 1A, 1C, and 2E; Tam and Loebel, 2007).

### Transcriptional Dynamics across the Differentiation Trajectory

**Figure 3.**
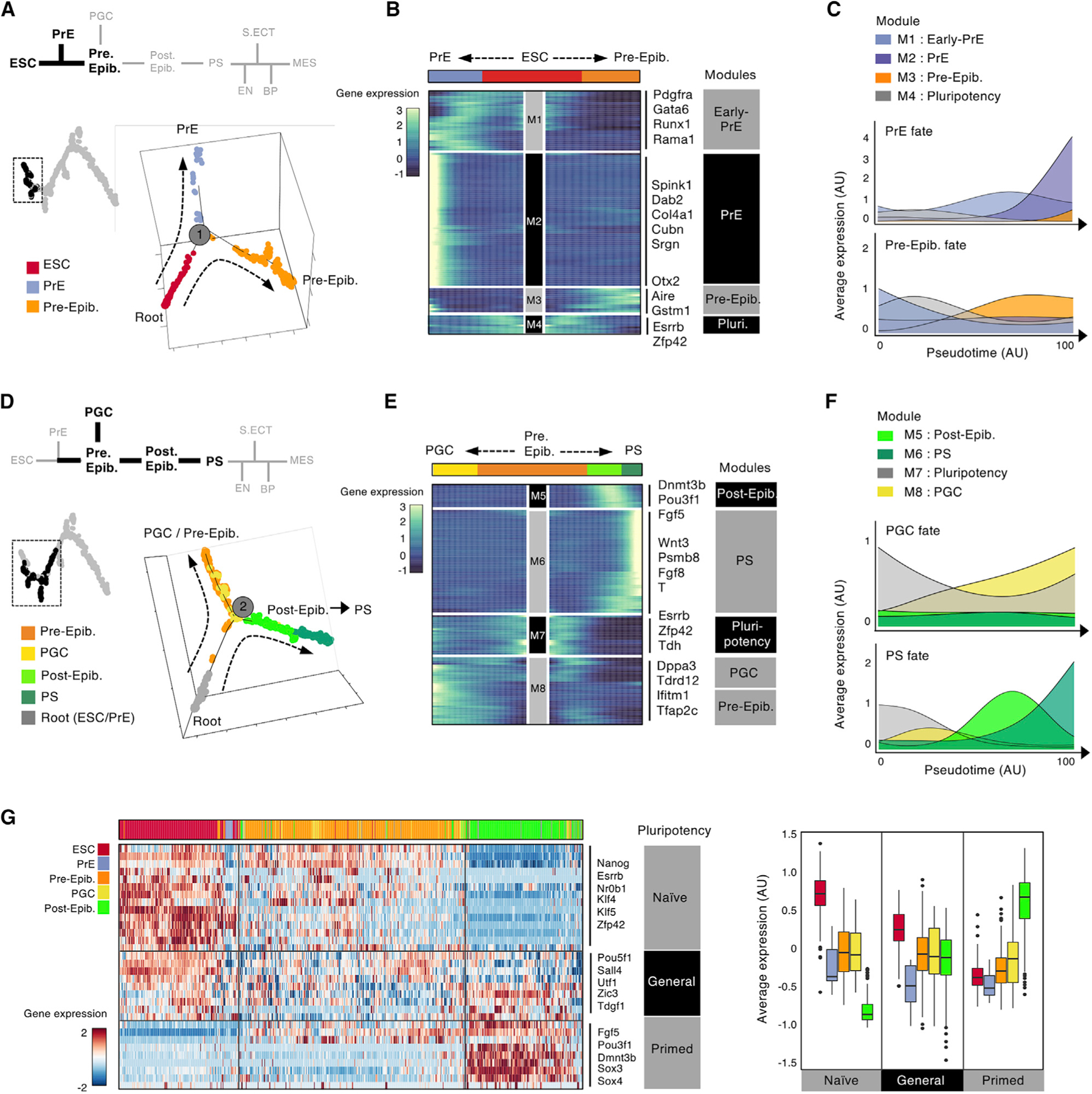
Transcriptional Dynamics across the Differentiation Trajectory (A) Schematic of the first major lineage bifurcation. ESCs differentiate into extraembryonic PrE-like or Pre-Epib cell states. The pseudotime trajectory was re-plotted for the 600 single-cell transcriptomes from days 0, 2, and 4. (B) Heatmap showing unbiased clustering of transcriptional programs for the 600 single-cell transcriptomes in (A). Cells are ordered by their pseudotime score radiating left (PrE branch, blue) and right (Pre-Epib branch, orange) away from the progenitor ESC population (center, red). The top differentially expressed genes for each cluster are annotated (p < 1e–10). (C) Graphs showing the average expression of each gene expression module from (B), plotted over pseudotime. (D) Schematic of the second major lineage bifurcation. Pre-Epib cells form PGC-like cells and Post-Epib cells (which, in turn, form the PS). The pseudotime trajectory was replotted for the 800 single-cell transcriptomes from days 2, 4, 6, and 8. (E) Heatmap showing unbiased clustering of transcriptional programs for the 800 single-cell transcriptomes in (D). Cells are ordered by their pseudotime score radiating left (PGC branch, yellow) and right (Post-Epib branch, lime green; forming the PS, dark green) away from the progenitor Pre-Epib population (center, orange). The top differentially expressed genes for each cluster are annotated (p < 1e–10). (F) Graphs showing average expression of each gene expression module from (E), plotted over pseudotime. (G) Heatmap (left) showing expression of naive, general, and primed pluripotency modules defined from Kalkan et al. (2017) in the single-cell transcriptomes from the annotated ESC, PrE, PGC, preimplantation epiblast, and postimplantation epiblast populations. Boxplots (right) quantify the gene expression modules from the heatmap. See also Figure S3 and Table S3.

The ordered graph of single cells generated by Monocle 2 implied at least six branch-points where a bipotent population bifurcates to two alternate branches. To discover the regulators of these cell fate decisions, we examined the dynamics of gene expression within each cell population bordering the trajectory branchpoints. The first bifurcation is segregation of ESCs into PrE or preimplantation epiblast-like cells. This occurs between days 0 and 4 in our time course (Figures 3A and S3A). This bifurcation approximates the inner cell mass (ICM), segregating into the PrE and epiblast at E4.5 (Boroviak et al., 2015; Mohammed et al., 2017; Tam and Loebel, 2007). We used hierarchical clustering of differentially expressed genes (Monocle 2, p < 1e–5) to define branch-specific transcriptional modules and gene expression patterns (STAR Methods). PrE cells closest to the branchpoint are defined by a gene expression module that includes expression of *Gata6* and *Pdgfra*, whereas a second gene module including *Col4a1*, *Cubn*, and *Srgn* is upregulated as differentiation along this branch continues (Figures 3B, 3C, and S3B). Conversely, the preimplantation epiblast-like population is defined by *Otx2* and *Aire* expression, which increases as cells differentiate along this branch. This bifurcation occurs before classical epiblast marker genes (e.g., *Dnmt3b* and *Fgf5*) are expressed in our time course (Figures 3E and 3F).

The second bifurcation involves specification of preimplantation epiblast-like cells into the PGC-like lineage or the postimplantation epiblast-like lineage. This occurs between days 4 and 6 in our time course (Figures 3D, S3C, and S3D). The presence of postimplantation epiblast-like cells suggests that this bifurcation approximates late peri-implantation. In our trajectory, cells differentiating along the PGC-like branch gain expression of PGC marker genes (*Dppa3*/*stella*, *Ifitm1*, and *Tdrd12*) and demethylation machinery (e.g., *Tet1* and *Tet2*). Conversely, cells differentiating along the postimplantation epiblast-like branch begin to strongly express epiblast marker genes (*Pou3f1/Oct6* and *Fgf5*) (Figures 3E, 3F, and S3D). Expression of these classic epiblast markers is strictly limited to the day 6 time point. Cells along the postimplantation epiblast branch also upregulate DNA methyltransferases, consistent with increased DNA methylation in this lineage *in vivo* (Lee et al., 2014). As cells continue in pseudotime along the postimplantation epiblast branch, they begin to express PS marker genes (*Wnt3a* and *Fgf8*) and sharply decrease expression of epiblast markers (Figures 3E, 3F, and S3D).

Implantation *in vivo* leads to a switch in epiblast cells from naive to primed pluripotency (Hackett and Surani, 2014; Mohammed et al., 2017; Nichols and Smith, 2009). We collated general, naive, and primed pluripotency modules (Kalkan et al., 2017) and hierarchically clustered cells from days 0–6 by their expression of these 3 gene modules (Figure 3G). We observed a similar switch in pluripotency state between preimplantation and postimplantation epiblast-like cells in EBs (Figure 3G). In EBs, preimplantation epiblast-like cells expressed naive and general pluripotency modules. In contrast, postimplantation epiblast-like cells expressed primed and general pluripotency modules (Figure 3G), with expression of all pluripotency factors sharply decreasing with increased pseudotime along the postimplantation epiblast branch toward PS-like cells (Figures 3E and 3F). *In vivo*, PGCs arise from the primed postimplantation epiblast at E6.5 and subsequently regain naive pluripotent status (Ohinata et al., 2009). In EBs, PGC-like cells appear to maintain their naive status and instead arise from the naive preimplantation epiblast-like population (Figure 3G).

Our graph-based trajectory next maps the progression of postimplantation epiblast-like cells to become the PS and subsequent branchpoints that specify the alternate germ layers and BPs. The trajectories and order of events are broadly consistent with known features of germ layer development (Figures S3E–S3M). In addition, the expression of marker genes at these later branchpoints closely resembles that seen *in vivo*. Briefly, as cells progress in pseudotime along the trajectory, PS cells first branch to form the EN and express classic EN markers such as *Foxa2*, *Gata4*, and *Dpp4*. Next the trajectory bifurcates to form the S. Ect (Surface Ectoderm), and cells express classic surface ectodermal markers, such as *Sox11*, *Col2a1*, and *Tlx1*. However, we caveat this inference on S. Ect with the observation that our EB derivation conditions favor MES (Keller, 2005) and may not faithfully recapitulate S. Ect trajectories. Finally, the trajectory bifurcates again to form the MES and BP terminal branches. The MES population is defined by class mesodermal markers such as *Postn*, *Nrp1*, and *Igfr2* and the BP population by *Tal1*, *Cdh5*, and *Esam* (Gritz and Hirschi, 2016; Keller, 2005).

Thus, unbiased analysis of single-cell transcriptomes for developing EBs reveals a spectrum of cell states and trajectories that recapitulate key features of preimplantation development and early embryogenesis. Our data support the value of this *in vitro* system for modeling and functionally interrogating early developmental programs and specification events and their epigenetic determinants.

### The Recombination-Based System Barcodes Cells in a Defined Temporal Window

We next sought to validate the trajectory architecture implied by the ordered graphs of single-cell transcriptomes. We initially explored genetic recording systems based on barcodes that evolve over cell divisions (e.g., Bhang et al., 2015; Chan et al., 2019; Frieda et al., 2017; Kalhor et al., 2017; Pei et al., 2017) but were unable to generate sufficient diversity in the narrow temporal window of our pre-gastrulation model. We therefore established a barcoding system that could be “timestamped” by Cre induction.

**Figure 4.**
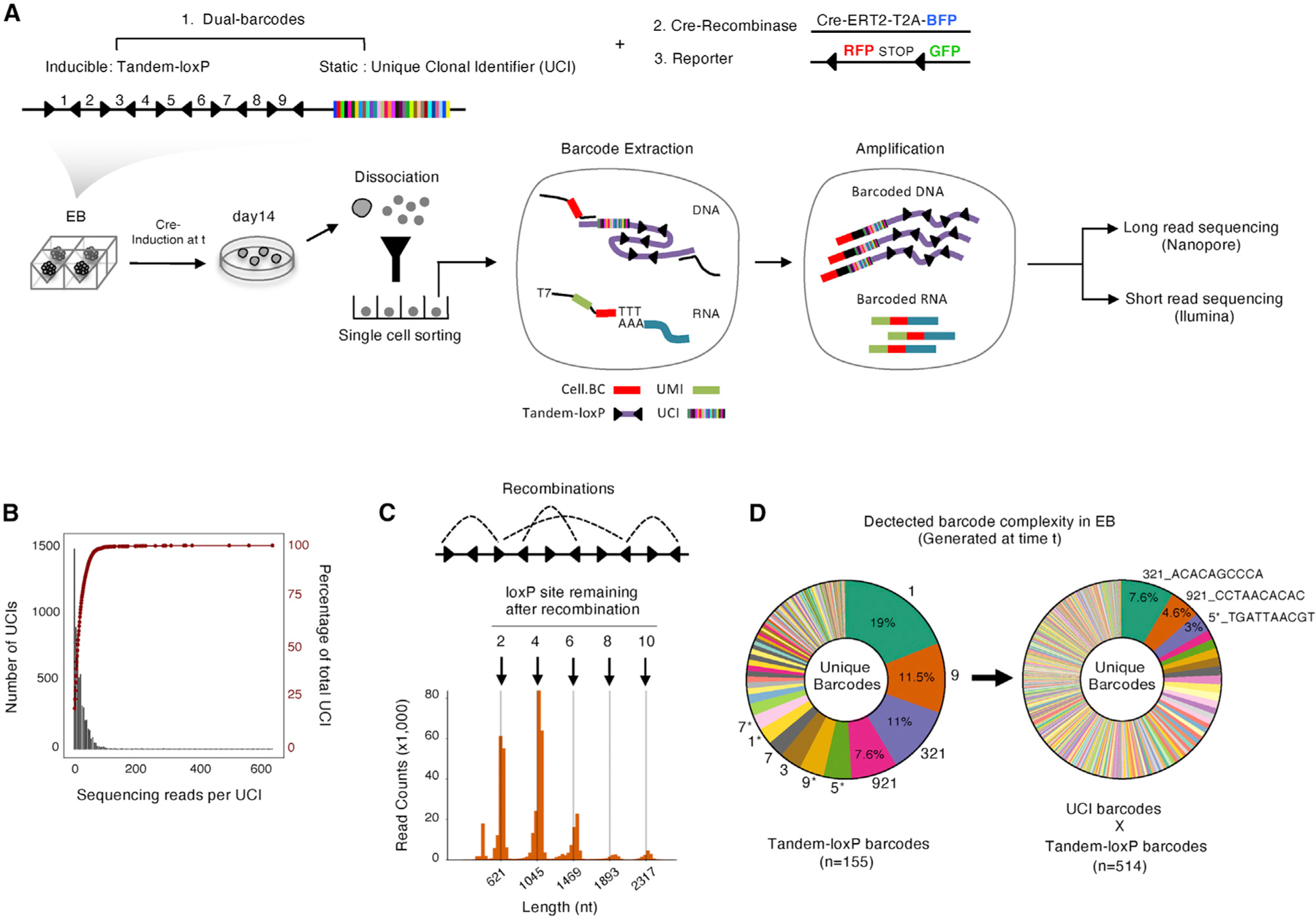
The Recombination-Based System Barcodes Cells in a Defined Temporal Window (A) Illustration depicting procedures for acquiring the transcriptome and timestamp barcode information from each single cell. ESCs are engineered to express an inducible Cre (Cre-ERT2-T2A-BFP), a Cre reporter (lox-RFP-STOP-lox-GFP), and a timestamp barcoding system containing a static barcode (N^10^ nucleotides) and an inducible tandem-loxP barcode (E2-crimson-tandem-loxp-UCI). ESCs are sorted into culture plates based on reporter expression (RFP, BFP, and E2-crimson) (as in Figure 1A). Recombination of the tandem-loxP sequence is induced by addition of Cre-ERT2. At the end of the 14-day time course, cells are harvested. The timestamp barcode is amplified using targeted primers and sequenced using Oxford Nanopore long-read sequencing. The transcriptome is profiled as before (STAR Methods). (B) The black histogram shows barcode distribution calculated from NGS data. The red cumulative frequency plot on the same plot shows that that 95% of barcodes are detected with 50 sequencing reads per UCI, consistent with observations from other barcoding studies (Bhang et al., 2015). (C) Barplot showing the length distribution of long sequencing reads after Cre-ERT2 induction. The tandem-loxP sequence contains 5 converging pairs of loxP sites with 9 spacer sequences, making an intact total of 2,317 bp. The full recombined product yields a 621-bp fragment. (D) Pie charts displaying the number of unique loxp recombined barcodes (left) and the number of unique loxP recombined barcodes+UCI (right). 5,000 static UCIs and a total of 155 achievable, temporally controlled tandem-loxP barcodes yield 514 unique barcodes in EBs after applying strict filtering criteria (Figures S4D–S4G; STAR Methods). See also Figure S4 and Tables S1 and S2.

We adapted the Polylox framework (Pei et al., 2017) with long-read nanopore sequencing (Figure 4A). We first cloned a cassette containing 10 tandem LoxP sites. Although Cre induction could theoretically generate 1.8 million possible recombined LoxP arrangements (Pei et al., 2017), we observed a strong bias for a relatively limited set of recombination events (Figure 4D, left). We therefore flanked the 10 tandem LoxP sites with a static barcode of 10 random nucleotides, which we call the unique clonal identifier (UCI), to ensure adequate complexity for lineage tracing (Figure S4A). We collectively refer to the temporal (LoxP) and static (UCI) barcodes as the timestamp cassette. Importantly, this cassette is read out from the genomic DNA in our strategy and therefore would not be compromised by silencing during differentiation.

We integrated the timestamp cassette, a LoxP-RFP-STOP-loxP-GFP (Cre reporter cassette), and a separate Cre-ERT2 construct into ESCs at a low MOI (<0.1) (Figure 4A). In an initial pilot, we induced recombination on day 0 (ESCs) by addition of tamoxifen for 30 min, followed by rapid washout (STAR Methods) and let the EBs differentiate for 14 days as above. We modified the CEL-seq2 protocol to amplify the mRNA and the DNA timestamp cassette from the same single cells (STAR Methods). This amplification procedure yielded cDNAs appended to cell-identifying barcodes (CBs) and the timestamp cassette, also appended to CBs. We then sequenced the cDNAs using Illumina sequencing and the timestamp cassettes (~2.5 kb) by long-read nanopore sequencing (Figure S4B). After we recovered a relatively uniform distribution of approximately 5,000 UCIs from a library (Figures 4B and S4C), we detected the tandem loxP barcode with the expected lengths and recovered 155 unique recombination outcomes from the 10 LoxP cassettes from a total of 4,224 cells on day 14 (Figures 4C, 4D, and S4D–S4G). When combined, the LoxP recombination and UCIs resulted in detection of 514 unique timestamp barcodes (Figure 4D), comparable with other barcoding technologies and a 5-fold increase over the original Polylox strategy (Figures S4H and S4I). These benchmarking experiments indicate that the combination of the timestamped LoxP barcode and the static UCI barcode should provide sufficient complexity to uniquely mark cells in EB time courses starting from roughly 1,000 ESCs.

### Timestamp Barcodes Support Inferred Lineage Relationships in EBs

**Figure 5.**
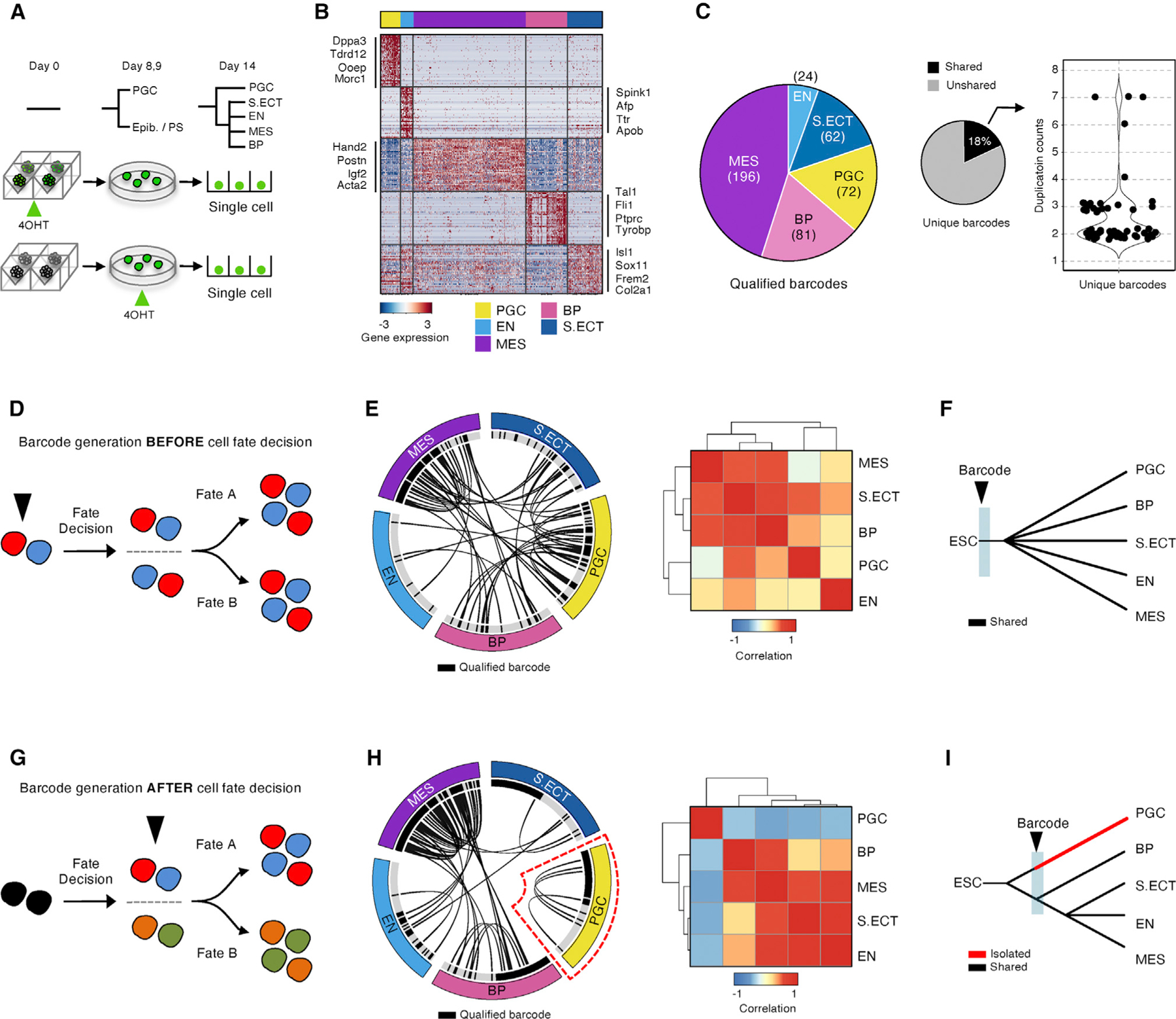
Timestamp Barcodes Support Inferred Lineage Relationships in EBs (A) Illustration depicting the experimental design for timestamp barcode generation. We initiated recombination of tandem-loxP barcodes at two time points: ESCs (day 0) and after expression of postimplantation epiblast marker genes (days 8/9). Cells were harvested on day 14 and processed for scRNA-seq and barcode detection as in Figure 4A. A total of 4,028 cells from 11 EBs passed QC. (B) Heatmap showing unbiased clustering of meta-modules based on the top 10 differentially expressed genes from 4,028 single-cell transcriptomes from the experiment in (A). Cells are color coded by cell-state annotations as in Figures 1 and 2. Lineage identity for each single cell was assigned as in Figure 1 and validated using correlations to *in vivo* datasets (Figure S5C) and using a machine learning classifier trained on data from Figure 1 (Figures S5A and S5B). Key marker genes of the major embryonic populations are highlighted. (C) Pie chart showing the number of timestamp barcodes identified in each lineage (left). 18% of the identified barcodes are shared across different lineages (center). The violin plot indicates how many times each shared barcode is counted in different cells. (D) Schematics showing the expected outcome of barcode generation in ESCs. Theoretically, all descendent lineages would share barcodes. (E) Linkage plot (left) showing the observed linkage map of cells when barcodes were generated in ESCs. Each connecting line represents 2 cells that possess an identical barcode (loxp+UCI). All major lineages are connected by multiple barcodes. The heatmap (right)shows correlation scores of detected barcodes over lineages. (F) Tree schematic depicting the shared origin of lineages when barcodes are generated in ESCs. (G) Schematic showing the expected outcome of barcode generation on day 8/9. Theoretically, descendants of lineages that are already distinct on day 8 would not share barcodes. (H) Linkage plot (left) showing the observed linkage map of cells when barcodes were generated on day 8/9. Each connecting line represents 2 cells that possess an identical barcode (loxp+UCI). All major lineages are connected by multiple barcodes, except the PGC-like lineage, which is distinct. The heatmap (right) shows correlation scores of detected barcodes over lineages. (I) Tree schematic depicting the distinct origin of lineages when barcodes are generated on day 8. See also Figure S5 and Table S4.

We next explored the potential of the timestamp system to validate lineage relationships in EBs. We generated EBs and induced recombination by exposing them to tamoxifen for 30 min at a time point corresponding to peak postimplantation epiblast marker gene expression (days 8–9). In a side-by-side control, we induced recombination on day 0 (ESCs) of the EB time course. We harvested cells on day 14 and performed parallel transcriptomics profiling and long-read DNA sequencing to retrieve the expression profiles and timestamp barcodes, respectively (Figure 5A). We acquired high-quality transcriptomics data for 4,224 single cells from a total of 11 EBs (STAR Methods).

We identified 5 distinct cell populations on day 14 that corresponded to the terminal branchpoints in our initial trajectory: EN, S. Ect, MES, BPs, and PGC-like cells (Figure 5B), with the expected absence of the extraembryonic PrE, which is lost by day 14. In addition, all major lineages were present in each single EB (Figures S6A and S6B). For consistency of lineage annotations, we used a random forest machine learning algorithm to classify cells from this experiment by their similarity to the cell states annotated in the original time course (Figures 1, S5A, and S5B; STAR Methods). The classifier performed well with PGC, EN, MES, and BP populations (Figure S5B), and correlation analysis confirmed the consistency with embryonic tissue (Figure S5C). Notably, the new dataset contained 20 times more day 14 cells than the original time course, which allowed us to also distinguish erythroid and myeloid-like cells within the BP-like population (Figure S5D).

We next recovered the timestamp barcodes from the long sequencing reads. Integration of the recombined loxP sequence, the UCI, and the cell barcode enabled us to distinguish a timestamp barcode for a total of 3,331 cells. We then filtered out cells that had a low-complexity, highly represented timestamp barcode (frequency, >0.005) (Figures S5E and S5G; STAR Methods). We also excluded cells with low-confidence lineage assignments (Figure S5F). Our final dataset consisted of 435 cells with high-confidence timestamp barcodes and transcriptome-based lineage assignments (Figures 5C and S5H).

Our inferred trajectory based on transcriptome data indicated that preimplantation epiblast-like cells were no longer present on days 8–9, when the timestamp barcodes were diversified by recombination. Thus, barcode recombination occurred after the major bifurcation of preimplantation epiblast-like cells to form postimplantation epiblast-like cells and the PGC-like lineage (Figure 3D), and after expression of the naive pluripotency module. When we examined the distribution of timestamp barcodes among cells harvested on day 14, we found that many barcodes were shared among the EN, S. Ect, and MES lineages but that PGC-like cells harbored a distinct set of barcodes (Figures 5G–5I). We validated these lineage relationships by varying confidence thresholds for barcode assignment (Figure S5J) and by observed-to-expected enrichment analysis (STAR Methods; Figure S5L). In stark contrast, in the control experiment in which recombination was induced on day 0 (ESCs), timestamp barcodes were shared by all populations (Figures 5D–5F and S5I). These data strongly suggest that the PGC-like cell state in EBs is specified before the postimplantation epiblast marker genes are expressed, and derives from the preimplantation epiblast-like cells.

This barcode recombination experiment also provided insight into the BP-like cells identified in day 14 EBs. The myeloid-like BP cells shared barcodes solely with the MES, consistent with MES derivation (Figures S5D and 5G–5I; data not shown). However, the erythroid-like BP cells harbored a limited distinct set of barcodes, suggesting that this population may be specified prior to the day 8–9 recombination event (Figures S5D and S5K). Notably, these erythroid-like cells also expressed the embryonic globin genes *Hba-x*, *Hbb-y*, and *Hbb-bh1*, potentially consistent with primitive hematopoietic cells derived from the yolk sac. We stress that this interpretation is caveated by the limited number of barcoded BP cells detected. However, the data do support MES derivation for myeloid-like BP cells in EBs and raise the possibility of distinct early embryonic derivation for erythroid-like cells.

These data and analyses provide insights into the differentiation trajectories of alternate EB lineages and demonstrate the unique advantages of our temporally controlled recombination-based barcode system for tracing lineage relationships when the number of cell divisions is limited. This recombination system is particularly well suited to EBs and other rapid differentiation systems.

### DNA Methylation Drives Cell Fate Choice in a Tight Developmental Window

PGCs arise *in vivo* in the postimplantation epiblast at the emergence of primed pluripotency (Ohinata et al., 2005). This switch from naive to primed pluripotency during implantation is concomitant with a striking global increase in DNA methylation (Argelaguet et al., 2019; Lee et al., 2014; Seisenberger et al., 2012; Tam and Loebel, 2007). In contrast, our experiments above suggest that, in EBs, PGC-like cells arise from preimplantation epiblast-like cells that still retain a naive pluripotent state. We therefore sought to understand the epigenetic determinants that underlie the fate choice between PGC-like and primed post-implantation epiblast-like cells in EBs.

**Figure 6.**
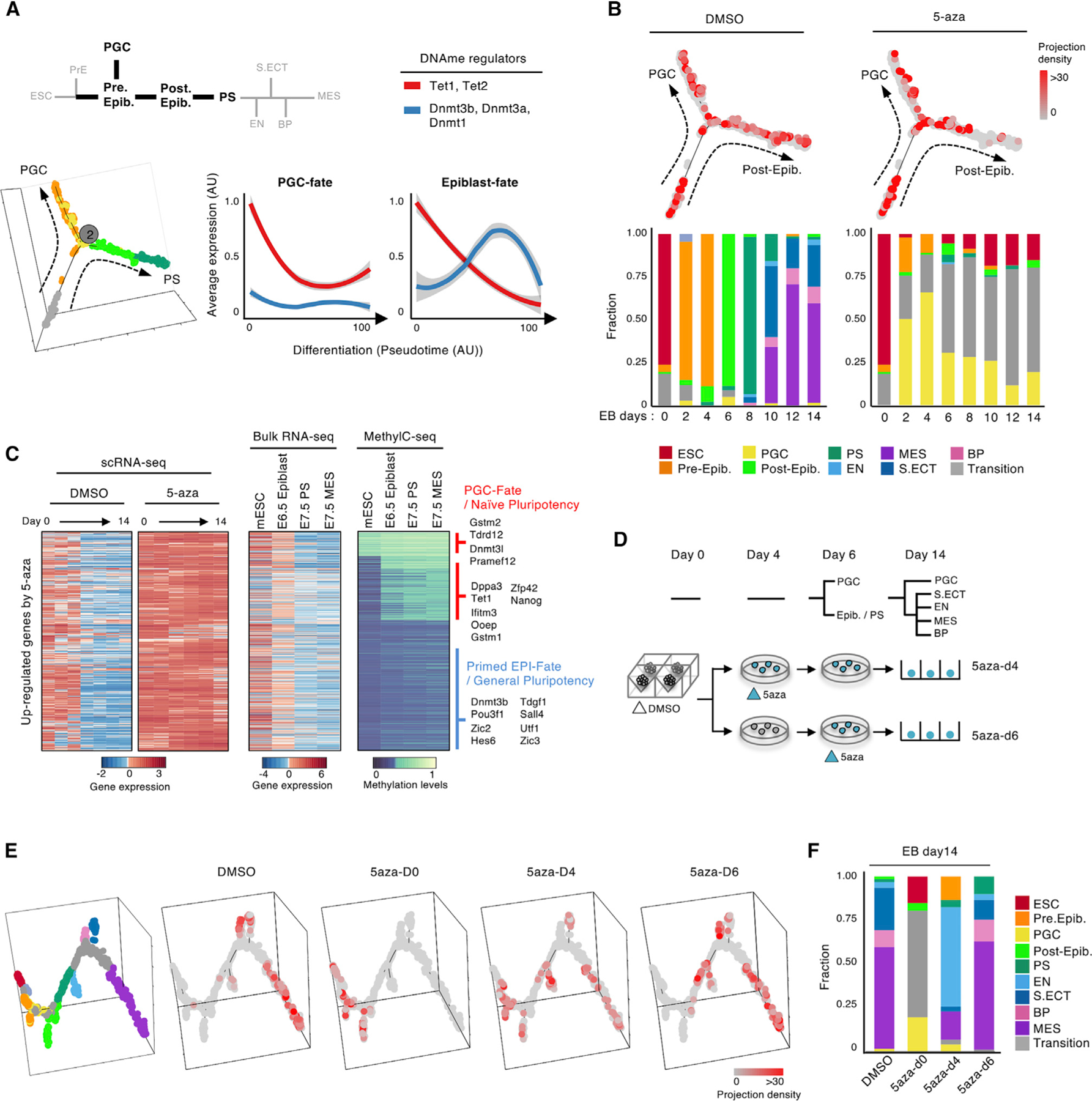
DNA Methylation Drives Cell Fate Choice in a Tight Developmental Window (A) Top: schematic representing the lineage trajectory. Bottom left: reproduction of the pseudotime trajectory plot from Figure 2D, depicting the branchpoint between Pre-Epib cells (orange), PGC-like cells (yellow), and Post-Epib cells (lime green; which become the PS, dark green). Bottom right: plots showing average expression of the DNA methylation and demethylation machinery as a function of pseudotime along each lineage branch, with 0 being the branchpoint. (B) EBs were treated with 5-aza and spontaneously differentiated to day 14. DMSO control or 5-aza-treated cells are projected (red points) on the Monocle 2 trajectory plot of the branchpoint from A (top). Stacked bar plots depict the proportion of each lineage at each time point (bottom). Lineages were assigned as in Figures 1 and 5. (C) Heatmap (left) showing the top upregulated genes after 5-aza treatment of EBs. The heatmap (center) shows that the same genes are downregulated during differentiation *in vivo* (GEO: GSE76505; Zhang et al., 2018). Another heatmap (right) shows that the promoters of the same genes become methylated during differentiation *in vivo* (GEO: GSE76505; Zhang et al., 2018). (D) Illustration depicting the experimental design for perturbation of DNA methylation at different time points. We treated cells with 5-aza on days 0, 4, and 6 and collected EBs for single-cell transcriptomics on day 14. (E) 5-aza-treated cells and DMSO-treated control cells as in (D) are projected on the EB trajectory shown in Figure 1B. (F) Stacked bar plots depicting the proportion of each lineage at each time point from the experiment described in (D) and (E). Lineages were assigned as in Figures 1 and 5. See also Figure S6 and Table S3.

As cells progress in developmental pseudotime from the preimplantation epiblast-like state toward the postimplantation epiblast-like state, they increase DNA methyltransferase expression and decrease DNA demethylase expression. Conversely, as cells progress in pseudotime toward the PGC-like lineage, DNA methyltransferase expression remains low, whereas DNA demethylase expression increases modestly (Figure 6A). The transcriptional state of these EB-derived PGC-like cells more closely resembles that of the preimplantation epiblast than the postimplantation epiblast (Argelaguet et al., 2019), and they also have lower global methylation levels (Figures S2G and S6J). We therefore hypothesized that a relative paucity of DNA methylation promotes PGC-like specification from preimplantation epiblast-like EB cells.

To test this, we introduced the hypomethylating agent 5-azacytidine (5-aza; 100 nM) over the full course of EB formation (STAR Methods). We performed single-cell RNA-seq (scRNA-seq) at multiple time points and assigned cell lineage identities as in Figures 1 and 5 (Figures S6C–S6F). Treatment with the hypomethylating agent resulted in a remarkable shift toward the PGC-like lineage (Figures 6B, S6G, and S6H). Although ~2% of cells in control EBs were PGC like, this lineage accounted for a full ~30% of cells after 5-aza treatment (Figure 6B). Further more, we observed complete absence of germ layers under the treated condition, with all cells approximating a naive state of pluripotency (Figures 6B, S6D, S6E, and S6G).

Genes significantly (t test, p < 1e–3) upregulated upon 5-aza treatment in EBs tended to be repressed in the epiblast, PS, and MES lineages *in vivo* (Figure 6C; Zhang et al., 2018). Moreover, the promoters of PGC-specific (e.g., *Dppa3*, *Tet1*, *Gstm2*, *Trdrd12*, and *Dnmt3l*) and naive pluripotency (e.g., *Zfp42* and *Nanog*) genes become methylated in epiblast, PS, and MES lineages *in vivo* (Figure 6C; Zhang et al., 2018). These data suggest that DNA methylation is critical for repression of naive pluripotency genes and PGC programs. They are consistent with a model where the hypomethylated window associated with early preimplantation development is critical to maintain naive pluripotency and competence for PGC specification.

Finally, we precisely defined the time window in which DNA methylation is critical for PGC and postimplantation epiblast-like fate choice. We again induced hypomethylation during EB formation but, in this case, initiated 5-aza treatment on day 4 or day 6. We then harvested the cells on day 14 and performed scRNA-seq as described above (Figure 6D). We found that introduction of DNA hypomethylation on day 4, when naive preimplantation epiblast-like cells are still present, but before classic postimplantation epiblast or PS marker genes are expressed, modestly increased the PGC-like fraction to 4% and strongly skewed EBs toward EN/VE (Figures 6E, 6F, and S6K). In contrast, introduction of hypomethylation on day 6, after emergence of primed postimplantation epiblasts, had essentially no effect on lineage distribution (Figures 6E and 6F). Similarly, 5-aza treatment of serum-grown ESCs in the absence of EB differentiation conditions yielded fewer than 1% PGC-like cells (Figure S6L). These data further support the conclusion that DNA methylation in naive preimplantation epiblast-like cells favors the postimplantation epiblast-like state, in part by suppressing PGC-like transcriptional programs. When the primed pluripotent epiblast state is established, DNA methylation is no longer required to maintain lineage-specific transcriptional programs, and hypomethylation cannot reprogram these cells for naive pluripotency and competence for PGC programs.

## DISCUSSION

The highly choreographed lineage hierarchy of mammalian embryogenesis has been painstakingly characterized over several decades by marker gene analysis. Recent technological developments in single-cell transcriptomics and lineage tracing now enable characterization of cell states and transitions at unprecedented resolution. Here we used the EB organoid model to map and perturb transcriptional and epigenetic programs that underlie cell fate specification. We acquired a dense time course of scRNA-seq data over 14 days of EB differentiation and used hierarchical clustering, Monocle 2, and machine learning to infer cell states and lineage trajectories. We then adapted a timestamp genetic recording system to generate cell-specific barcodes in narrow temporal windows and validate key developmental branchpoints in this highly dynamic system. We identify early specification of a PGC-like cell state from cells that closely resemble naive preimplantation epiblast-like cells. We find that this critical specification event is tightly controlled by DNA methylation, which silences PGC programs in a precise temporal window in preimplantation epiblast-like cells. Our study provides insight into pre-gastrulation cell fate decisions and a set of tools for mapping lineage relationships in rapidly differentiating systems.

Single-cell transcriptomic and pseudotime temporal ordering of cells is a powerful approach to infer lineage relationships, which then require validation by direct lineage tracing approaches. We integrated a suite of technologies to map and validate cell state transitions in spontaneously differentiating EBs. We used scRNA-seq to map ~6,000 genes per cell. This high-transcriptome coverage increases confidence in lineage assignments, particularly for cells in transitional states. We also innovated a timestamped barcode system for lineage tracing that could be rapidly recombined, as opposed to CRISPR-based barcodes, which require many cell divisions to evolve complexity (Chan et al., 2019; Kalhor et al., 2018; Ludwig et al., 2019; McKenna and Gagnon, 2019; Pei et al., 2017). We read out the transcriptome and timestamped barcode from the same single cells by combining scRNA-seq and long-read DNA sequencing. A key innovation was inclusion of an additional UCI that enabled us to identify and control for overly frequent recombination events and, thus, reduce false positives. Our method is readily extensible to other rapidly differentiating systems or single-cell sequencing technologies.

Our trajectory analysis and lineage tracing demonstrate that EB-derived PGC-like cells arise from a naive pluripotent preimplantation epiblast-like state. This is in seeming contrast to the emergence of PGCs from the primed pluripotent postimplantation epiblast at E6.25, which is almost concomitant with emergence of the PS (Ohinata et al., 2005; Yamaji et al., 2008). This may be a limitation of the EB model, which lacks spatial cues of the developing blastocyst. However, recent studies have elegantly described a continuum of pluripotent states in the developing epiblast, including an intermediate or “formative” state of pluripotency from which unipotent germ cells arise (Cheng et al., 2019; Kalkan et al., 2019; Kolodziejczyk et al., 2015; Messmer et al., 2019; Rostovskaya et al., 2019; Smith, 2017). Indeed, the preimplantation epiblast-like cells in EBs closely resemble this intermediate/formative pluripotency state (Figure S6E). Thus, naive preimplantation epiblast cells in the EB could already be primed for PGC formation without the spatial cues of the postimplantation blastocyst environment.

Last, our data provide specific insights into the mechanisms by which DNA methylation directs lineage fates in the preimplantation development. We find that promoter methylation suppresses naive pluripotency and PGC transcriptional programs in EB preimplantation epiblast-like cells and favors postimplantation and primed pluripotency programs. Taken together with the single-cell transcriptomic data, our study supports the hypothesis that naive preimplantation epiblast cells are epigenetically primed for different cell fates by their differential DNA methylation. Indeed, pluripotent epiblast cells *in vivo* are also primed for ECT fates as early as E4.5 (Argelaguet et al., 2019). Our data also suggest that DNA methylation is only critical for lineage specification in the naive preimplantation epiblast-like state. When the primed postimplantation epiblast transcriptional state is set, all other lineage-specific transcriptional programs can still be derived in the absence of DNA methylation. Induction of hypomethylation in these cells does not cause them to regain naive pluripotency and competence for PGC formation, consistent with increased importance of other chromatin- and transcription-based mechanisms in reinforcing postimplantation lineage identity (Nicetto et al., 2019; Wang et al., 2018). Taken together, our data suggest that the observed increases in DNA methylation in the epiblast *in vivo* are likely to influence the fate of naive preimplantation epiblast cells prior to emergence of primed pluripotency, when other epigenetic mechanisms gain prominence. Although our data emphasize a role of hypomethylation in facilitating PGC cell specification, they do not distinguish whether PGC precursors maintain a hypomethylated genome within the preimplantation epiblast or proceed through a transient hypermethylated state.

In summary, we report a developmental trajectory and single-cell transcriptional atlas for an organoid model of pre-gastrulation embryogenesis. We nominate and validate transcriptional and epigenetic regulators of key fate decisions. We also provide a toolkit for lineage tracing that is compatible with rapidly differentiating biological systems. Taken together, these data and tools provide a rich resource for charting developmental hierarchies, cell fate decisions, and the factors that regulate fate choice.

## STAR★METHODS

### RESOURCE AVAILABILITY

#### Lead Contact

Further information and requests for resources and reagents should be directed to and will be fulfilled by the Lead Contact, Bradley E. Bernstein (bernstein.bradley@mgh.harvard.edu).

#### Materials Availability

Plasmids generated in this study will be deposited in Addgene.

#### Data and Code Availability

The accession number for the sequencing data reported in this paper is GEO: GSE140890. We also utilized published datasets GSE76505 and E-MTAB-2958. The code generated during this study is available from the corresponding author on request.

### EXPERIMENTAL MODEL AND SUBJECT DETAILS

We cultured mouse v6.5 ESCs with feeder cells (mouse embryonic fibroblasts (MEFs), Globalstem, GSC-6002G) in knockout DMEM media supplemented with 1% Pen Strep (Thermo, 15140163), 1% L-Glutamine (Thermo, 25030081), 1% Non-Essential Amino Acids (Thermo, 11140076), 15% FBS (GE, SH30070.02E), 0.004% 2-mercaptoethanol (Sigma, 6010), and 0.01% Leukemia inhibitory factor (LIF; Millipore, ESG1107). We replenished the media every day and cells were split every two days.

### METHOD DETAILS

#### Generation of timestamp-barcode system

We synthesized (Genscript) a DNA sequence containing tandem LoxP sites (Table S1), and ligated this sequence to the 3′ end of the E2crimson gene in a lentiviral vector engineered to express PuroR-T2A-E2crimson from the EF1a promoter (Table S1). We then synthesized a UMI DNA oligo (IDT, diluted to a 100uM) that includes the following elements: M13F-N(10)-HSV polyadenylation site-M13R. We PCR amplified this sequence using 0.5 ul of the UMI oligo, UMI-Adaptor-DigF/R primers (Table S2), and superFi DNA polymerase using the manufacturer’s guidelines (35 cycles, annealing at 55°C for 10 s, extension step for 5 s, 35 cycles in total). After amplification, 20ul of ExoSAP-IT (Affymetrix, 78200) was added and the reaction was incubated at 37°C for 15 minutes, followed by incubation at 80°C for 15 minutes. 2 separate reactions were performed for the UMI oligo, which were then combined and precipitated with sodium acetate (10% volume from a 3M solution) and isopropanol (1:1 volume). The solution was stored at −20°C for 30 minutes, spun down at 15000 Gs for 15 minutes, washed with ethanol, and then resuspended in 20 ul of water. The amplified DNA was digested with BamHI and SalI overnight. We then ligated the UMI sequence into a lentiviral vector engineered to express PuroR-T2A-E2crimson and the loxP sequence from the EF1a promoter at the 3′ end of the loxP DNA (Table S1). We used a ligation ratio of 3:1 (insert to vector, 100ng vector) for 1 hour at room temperature, and then transformed each of 10 tubes (50 ul) of chemically competent NEB stbl cells with 2ul of the ligation reaction. We then grew the bacteria on LB agar plates at 30°C overnight. The next day, the colonies (~40,000) were scraped off using a glass spreader, and inoculated into 2 cultures of LB each containing 2 Liters with ampicillin (100 ug/ml), shaken for 4 hr at 37°C, and subjected to a maxiprep plasmid DNA extraction.

#### Lentivirus generation and infection of ES cells

mESCs were infected at an MOI of 0.1 for CreERT2-BFP, and Cre reporter vectors (Table S1). The p-EF1a-PuroR-T2A-E2Crimson-tandemLoxp-UMI vector was infected with an MOI of 0.1 to ensure only 1 integration per cell. We added polybrene at a final concentration of 1 ug/ml for all infections then selected cells using antibiotics (puromycin) and performed FACS for E2Crimson positive cells. After integration of the timestamp-barcode lentiviral system, we performed FACS for RFP+/GFP−/E2-crimson+/BFP-mid to ensure the expression of all plasmid and tightly controlled inducible system, and then immediately started EB differentiation.

#### EB differentiation

We incubated mESCs cultured on irradiated feeder MEFs together in ESC dissociation buffer (500ul of 0.5M EDTA and 0.9g of NaCl in 500ml Calcium/Magnesium free PBS) for 20 minutes at 37°C. We next detached mESC colonies from MEFs by gentle pipetting and dissociated ESCs to single cells by incubating with Accumax for 5 minutes at 37°C. To ensure removal of MEFs, we incubated single cell suspensions of ESCs on a 100mm tissue culture dish for 40 minutes at 37°C. We then collected the supernatant which contained mESCs only. We seeded a 1,200 well micro-well plate (STEMCELL Technologies, 27945) with 1.2 million ESCs in EB media (mESC media without LIF), to obtain a density of ~1000 cells per well. This approach allowed us to obtain morphologically consistent EBs controlling for spontaneous differentiation which can be altered by EB size and shape (Koike et al., 2007). For EB differentiation with inducible barcoding experiments, we seeded FACS sorted RFP+/GFP−/BFP^mid^ cells to ensure Cre expression and remove unwanted recombination by un-controlled CreERT2 activation.

After 4 days of EB differentiation in the micro-well plate, we collected EBs and transferred them to 100mm Petri dishes and pursued spontaneous differentiation for another 10 days. EB media was changed every day (micro-well plate) or every other day (Petri dish) during EB differentiation.

To determine the optimal differentiation time window to detect spontaneous differentiation, we extracted bulk RNA from EBs at every 2 days and measured mRNA expression levels of lineage specific marker genes (ESC: Pou5f1 and Nanog, Post-Epib.: Dnmt3b and Fgf5, Primitive Streak (PS): T and Wnt3, Ectoderm: Pax6 and Prom1, Endoderm: Sox17 and Foxa2 and Mesoderm: Pdgfra and Kdr.) ESC maker genes were gradually decreased through 14 days and the 3 germ layer (ectoderm, endoderm, and mesoderm) markers were increased along differentiation (day10~14). The oscillating expression of Post-implantation Epiblast and PS markers had a peak at day 6 and day 8, respectively.

#### Timestamp barcode induction

To generate barcodes at specific time points, we treated cells containing the timestamp loxp cassette, and the CreERT2 construct with 25nM of 4-Hydroxytamoxifen (4OHT Sigma, H7904). Cells were incubated with 4OHT for 30 minutes (day 0 induction) or 1 hours (days 8–9 induction). Cells were then washed three times with PBS and then cultured to resume differentiation. Recombination events were confirmed by FACS for RFP−/GFP+ (Cre reporter).

#### Cell sorting

We dissociated EBs using Accumax (Sigma, A7089) or Trypsin (invitrogen). Cells were incubated with the dissociation enzyme for 20 minutes at 37°C with frequent trituration. We then washed the cells with PBS containing 2% FBS and filtered the cells through a 30um cell strainer (Stem cell technology). The resulting cell suspension was incubated with live cell count dyes (LIVE/DEAD aqua; ThermoFisher, L10119) for 15 minutes on ice and washed with PBS containing 2% FBS. To check for spontaneous differentiation of EBs to the 3 germ layers, cell suspensions were further stained using antibodies that recognize germ layer markers for 30 minutes on ice: Pdgfra or Kdr for Mesoderm, Cxcr4 for Endoderm, Prom1 for Ectoderm (Table S3). FACS was performed on a Sony SH800 sorter.

To perform FACS for single cell sequencing, live cells were sorted into single wells of 384 well plates that were preloaded with 1.8 μL of distilled water involving 7.5 μg/μl of unique T7-polyA RNA barcoded adapters (RNA-adaptor; Table S2) and 1.5mM of dNTP mix. Plates were stored at −80°C until processing.

For parallel single cell sequencing, Cre induced and subsequent loxP barcode generated each EB was isolated into a single well of 96 well plates at day 14 and then dissociated, stained, and washed within a well to ensure single EB preparation. E2Crimson positive live cells were sorted into the cell-barcode plate (384 well) as stated above.

#### 5-azacytidine treatment of EBs during differentiation

To perturb DNA methylation of EBs during differentiation, we treated EBs at day 0 (micro-well stage) of our differentiation time-course with 5-azacytidine (Sigma, A2385, 100 nM). We replenished 5-azacytidine with every media change throughout all 14 days of differentiation. We treated EBS with DMSO in parallel as a control. We collected EBs every 2 days for 14 days, dissociated them, and then performed single cell sorting. We performed scRNA-seq on 768 cells for both 5-azacytidine and DMSO treated EBs. To investigate when the PGC-like and postimplantation epiblast-like lineages are defined, we treated EBs with 5-azacytidine at day 4 or day 6, targeting either before or after postimplantation marker genes are expressed. We sustained treatment until day 14. EBs were then collected at day 14 and we performed scRNA-seq.

#### Single cell RNA sequencing

We collected EBs (~500) every 48 hr for 14 days, from the same plate of growing EBs, and we then applied CEL-seq2 method (Hashimshony et al., 2016) to profile single cell transcriptomes of time-coursed EBs. After freeze and thaw twice of FACS sorted single cell plates and incubation at 65°C for 5 minutes, we added a 1.2 μL of RT mixture (0.15 μL of SuperscriptII (Thermo, 18064–014), 0.15 μL of RNaseOUT (Thermo, 10777–019), 0.3 μL of 0.1M DTT, 0.6 μL of First strand buffer and 0.1% of IGEPAL (Sigma)) and performed a reverse transcription at 42°C for 1 hour, followed by heat inactivation at 70°C for 10 minutes. We next added a 10 μL of second strand synthesis mixture (0.35 μL of E.coli DNA polymerase I (Thermo, 18010–025), 0.09 μL of RNaseH (Thermo, 18021–071), 0.09 μL of E.coli DNA ligase (Thermo, 18052–019), 0.025 μL of 10mM dNTP mixture (Thermo, R0192), 0.25 μL of Second strand buffer (Thermo, 10812–014) and 6.72 μL of distilled water) and performed a second strand synthesis at 16°C for 2 hours. We then pooled 96 wells to a sample and purified it by 1.2X AMpureXP beads (Bechman, A63881). Purified samples were linearly amplified by IVT (MEGAscript T7 Transcription Kit (Thermo, AM1334)) at 37°C for 15 hr. Next, we added ExoSAP-IT (Thermo, 78200) and incubated samples at 37°C for 15 minutes to remove leftover primers. We then fragmented RNAs at 94°C for 3 minutes (200mM Tris-acetate, pH 8.1, 500 mM KOAc, and 150 mM MgOAc) and followed by adding a STOP buffer (0.5 M EDTA pH8). After -purification, amplified RNAs were reverse transcribed by random-hexamers and then further amplified by illumina adapters to generate sequencing libraries (High-Fidelity PCR Master Mix (NEB, M0531S); Table S2). All the scRNA-seq libraries were sequenced using the Hiseq2500 platform.

#### Parallel single cell sequencing

To process plates with single cells for parallel RNA and DNA sequencing, we first thawed the plates containing sorted cells, and then subjected each plate to another freeze-thaw cycle. We then performed reverse transcription by unique RNA adapters, followed by second strand synthesis. We then added 0.5 μL of proteinase-K (1.4 μg/μl, Thermo, EO0491) and incubated plates at 50°C for 1 hour, followed by a heat inactivation at 85°C for 20 minutes. Next, We added 1.5 μL of DNA amplification mixtures: 0.15 μL of Q5 Hotstart enzyme (NEB, M0493L), 0.3 μL of 10mM dNTP mix, 0.225 μL of distilled water, 0.075 μL of a forward DNA-primer (100 μM) and 0.75 μL of barcoded DNA adapters (10 μM), designed to encode an identical cell barcode to the RNA-adaptor used in the same well, to amplify both the static and inducible barcode region of genomic DNA in each well (Table S2). We then PCR amplified the barcode region: initial amplification by 5 cycles of 98°C for 15 s, 60°C for 30 s, and 72°C for 90 s, followed by 15 cycles of 98°C for 15 s, 72°C for 90 s. After purification of PCR products, we next performed IVT for RNA amplification, and then pooled all the wells. The mixture was then divided between scRNA-seq reactions (70%) and a DNA library for barcode detection (30%). For the RNA library, we performed a library construction by scRNA-seq protocol as above. To prepare the DNA library to readout the timestamp-barcode, DNA primers further amplified the DNA library: initial amplification by 5 cycles of 98°C for 15 s, 62°C for 30 s, and 72°C for 90 s, followed by 10 cycles of 98°C for 15 s, 72°C for 90 s (Table S2). The purified DNA library was subjected to a long read sequencing.

#### Long-read single cell sequencing

We prepared libraries for Nanopore sequencing per manufacturer’s guidelines (NBE_9065_v109). Amplicons containing fragments between 700 bp and 2500 bp were purified using a 1.0X AMPure XP bead cleanup, and library construction was performed using the SQKLSK109 (1D) Ligation Sequencing Kit (Oxford Nanopore Technologies, ONT) according to manufacturer’s instructions, with some modifications. Briefly, 100 ng purified DNA from a pool of barcoded single cells was subjected to end repair and dA-tailing using the NEB-Next Ultra II End-Repair/dA-tailing Module. Next, we performed a 1X volume AMPure XP bead cleanup and ligated nanopore barcodes to each sample using the 1D Native barcoding kit (EXP-NBD104 / EXP-NBD114) and the Blunt/TA Master Mix (NEB). After a 1X AMPure XP bead cleanup, equimolar amounts of each sample were pooled and nanopore sequencing adapters were ligated to the eluted DNA using the Quick T4 ligase (NEB). The final clean-up of the adaptor-ligated DNA was modified and performed with 0.5X AMPure XP beads. We used a total of 60 ng of the final library to load into a MinION flow cell. We sequenced each flow cell for 10–48 hours and obtained over 10 million reads per run. The computational analysis of long-read single-cell sequencing data is described below.

### QUANTIFICATION AND STATISTICAL ANALYSIS

#### Single cell RNA sequence extraction and alignment

Sequencing RNA libraries, passed through quality filter (cluster density, total yield, and per-cycle base quality), were then split by library barcodes using bcl2fastq (v.1.8.4) and default setting. A 6bp of cell barcodes and another 6bp UMI were in Read1 of 18bp reads. The extra bases were added to prevent misleading interrupted sequences by accident. The sequence of transcript was in Read2 of 36bp reads. We adopted CEL-seq2 pipelines (https://github.com/yanailab/celseq2) to process the single cell data. To demultiplex the data, we split Read2 into separate files based on the cell barcode from Read1 and attached UMI to Read2 metadata. Unclassified sequences were 0~20% of total reads in scRNA-seq and 0~40% of total reads from samples subjected to parallel sequencing. We then mapped the reads of each cell to a mouse reference genome (mm9) using Bowtie2 (v.2.3.4). Average mapped sequences were over 70% of total reads in scRNA-seq and over 50% in samples subjected to parallel sequencing. Finally, we identified and eliminated reads sharing the same UMI, and then generated an accurate molecule count for each gene followed by converting the number of UMIs into transcript counts. Average gene complexity was 6000 in scRNA-seq and 3000~4000 in the samples in which we sequenced both RNA and the timestamp barcode from gDNA sequencing. We used transcript counts as a digital gene expression matrix for downstream analysis. Resulting fastq files were deposited in GEO (GSE140890).

#### Single cell RNA data pre-processing

We applied the sequential steps of single cell processing pipelines (Seurat R-package, v.2.2) for QC, normalization, dimensionality reduction and clustering with the following modifications: In brief, we first discarded low quality cells with abnormal gene complexity (fewer than 2,000 or over 10,000; average count is 6,000), high proportion of mitochondrial genes (> 10%), and over 200,000 UMI counts. For samples subjected to the recording data (parallel DNA/RNA sequencing), we used a different threshold of these parameters (gene complexity: 1,000~8,000, UMI: over 20,000). We next normalized the data by total counts, multiplying scale factor (10,000), and log-transformation, and then scaled to zero mean expression and unit variance. For the EB data at the terminal differentiation time point, we further modeled the relationship between gene expression and cell cycle score (G2/M and S phase marker genes) and used the scaled residuals for downstream analysis to subtract cell cycle heterogeneity. We then selected highly variable genes based on variance mean ratio and applied principal component analysis (PCA) for dimensionality reduction. For batch correction of replicate datasets, we applied canonical correlation analysis (CCA) with separately normalized and scaled datasets to project each dataset into the maximally correlated subspaces by the canonical correlation vectors. We visualized the data by plotting the t-distributed Stochastic Neighbor Embedding (t-SNE) using top PCs or aligned CCs. To cluster cells, we then performed a shared nearest neighbor (SNN) for embedding cells and Louvain clustering for modularity optimization (resolution parameter 0.8).

#### Single cell timestamp-barcode extraction

We converted current-recording files (Fast5) of nanopore sequencing data to fastq files by Guppy (v.2.3.5). Resulting fastq files were deposited in GEO (GSE140890).

To identify the cell identity, the cell barcode (2×6bp = 12bp), together with its upstream (20bp) and downstream (24bp) sequence were used to map nanopore reads using minimap2. Each nanopore read is assigned to a cell barcode based on its highest mapping score.

To identify the Polylox barcode for each cell, minimap2 was used to map sequences of 9 different DNA blocks with loxP, WPRE_M13R_HSV and pBC00 to the nanopore reads that have cell barcodes detected as described above. We then assembled all the sequences into a full Polylox barcode based on their mapping position in the nanopore reads. Due to the sequencing error, truncation of sequencing and cross-contamination of cell barcodes, for all the reads carrying the same cell barcode, more than one Polylox barcodes were detected. Therefore, the frequency of each Polylox barcode among all the reads was calculated to determine the true Polylox barcode for each cell. We selected the most frequent barcode with additional filters: 1) The selected barcode should be detected in more than 30 reads. 2) The number of reads of selected barcodes should be much more than other barcodes detected and considered as an outlier in statistics (Figure S4E). 3) The selected barcode should be detected as full length with the flanking sequence of the WPRE_M13R_HSV and pBC00.

To identify UCI for each cell, we first mapped the upstream (M13F: 74bp) and downstream (WPRE_M13R_HSV: 104bp) of the UCI sequence to nanopore reads by minimap2. After having the position of M13F and WPRE_M13R_HSV sequence, we extracted the sequence between M13F and WPRE_M13R_HSV. Due to the sequencing error, we selected the most frequent UCI sequence with 3 additional filtering steps to ensure we selected the correct UCI sequence: 1) The selected UCI sequence should be detected in more than 30 reads. 2) The number of reads of selected UCI sequence should be much more than other UCI sequence detected and considered as an outlier in statistics. 3) The selected UCI sequence should be matched to the sequence from UCI plasmid.

With these criteria, we detected 514 unique timestamp-barcodes (Polylox and UCI combination) with high confidence (18% of total; Figure S5E).

#### Linkage map of lineages

The recombination bias of Polylox and uneven distribution of the UCI sequences results in overrepresentation of some timestamp-barcodes. To identify the lineage relationships between different cell types, we need to exclude high frequency barcodes that are likely to be shared by more than one cell at the beginning of the experiment. To estimate the frequency of each timestamp-barcodes, we calculated the frequency of UCI and Polylox barcodes separately in single EBs at day 14. As the generation of Polylox and the distribution of UCI barcodes are two independent events, we multiplied the frequency of UCI and Polylox barcodes to estimate the frequency of each timestamp-barcode at the beginning stage. Next, we applied frequency cutoffs 1/1000, 3/1000, 5/1000, 1/100, leading to different numbers of cells for downstream analysis. Different cutoff values lead to similar conclusions. With 5/1000 cutoff, we end up with 435 timestamp-barcodes (30% of detected barcodes) for the linkage map.

We then combined transcriptional profiles and extracted timestamp-barcodes for each single cell using the same cell barcodes available in both analyses. We connected cells with identical timestamp-barcodes, and assigned lineages to each cell by annotated cell clusters through parallely sequenced RNA profiles (Table S4). We used the circlize R package (v 0.4.5) to visualize the connections between lineages. To compare the overall relationship of cells based on their cell-of-origin, we selected all the shared timestamp-barcodes and counted the frequency of each barcode for each lineage. We also calculated the e pairwise spearman correlation between different lineages based on frequency of shared timestamp-barcodes.

#### Trajectory analysis

Among several trajectory algorithms, we adopted the graph-based, machine-learning algorithm, called Monocle2 (R-package v.2.2) (Qiu et al., 2017) since it allowed us to emphasize bifurcation of cell fate decisions over putative lineage branch points. We performed Monocle with the following modifications: we imported a normalized, scaled and batch-corrected gene-expression dataset generated by our Seurat analysis for consistency across analyses. For the separate trajectory analysis targeting only early and late branch points, we generated an individual dataset with cells from days 0–4, 0–8 and 8–14, and extracted highly variable genes for each data-set. To order cells, we projected a gene-expression dataset into a lower dimensional space and then applied a reversed graph embedding algorithm to learn the structure of the trajectory with unsupervised analysis, and then assigned a pseudotime to each cell based on the distance to the root. To resolve complex branching processes inferred by 3 germ layers from our initial analysis, we tested a default parameter of maximum dimension for total EBs, and then adjusted it to 3 for early or late EBs and 5 for total EBs. We visualized the trajectory in 3 dimensional spaces (rgl R-package, v.0.98), and then displayed all information of each single cell on the tree structure (Cell identity, EB days, and gene expression). Separate trajectory analysis targeting early and late branch points revealed 5 branch points through 14 days of EB differentiation.

#### Lineage annotation based on reconstructed trajectory

The trajectory of total EBs suggested several branches and stems as segments on the tree structure. To find differentially expressed genes (DEGs) per segment over other cells, we performed the Wilcoxon ranked test (Seurat) in early and late EBs, separately. Segments fewer than 5 cells were not included for DEG calculation. We filtered DEGs that greater than a minimum detection percentage (0.25) and a minimum fold change (logFC > 0.25). We then selected top 10 genes by a positive fold changes and ranked through lowest p value (P value < 1e-3). Using top 10 genes per each state, we hierarchically clustered cells by Pearson correlation of expression profiles of DEGs. We collapsed segments to 9 clusters by similar gene-expression patterns. We then compared the realtime and pseudotime variables and found a group of 36 cells that had unmatched real and pseudotime. We annotated these cells as arrested cells. To annotate clusters as corresponding embryonic lineages, we first investigated well-known marker genes (Table S3) of anticipated embryonic tissues. We identified developing embryonic lineages on cell clusters, such as Primitive Endoderm (PrE)-, Preimplatation Epiblast (Pre-Epib.)-, Postimplantation Epiblast (Post-Epib.)-, Primordial germ cell (PGC)- (“arrested”), Primitive Streak (PS)-, Definitive Endoderm (DE)-, Surface Ectoderm (S.ECT)-, Blood Progenitor (BP)- and Mesoderm (MES)-like cells, by distinctly expressed marker genes. Second, we cross-validated the expression of extracted DEGs in corresponding lineages of developing embryo (see method “Comparison the in vivo and in vitro RNA-seq data”). We then assigned the corresponding embryonic lineages on each cluster and displayed them on the trajectory.

#### Clustering and lineage annotation of parallel sequencing data

We clustered 4,028 cells using highly variable genes based on variance mean ratio and then displayed expression profiles of the top 30 differentially expressed genes (DEGs) from 5 clusters including 2 collapsed clusters that express erythroid and myeloid marker genes as BP. We assigned lineages to clusters by marker genes previously used to annotate lineages (Table S3) and validated their consistency to timed EB data by lineage prediction scores based on Random Forest classifiers. Assigned lineages were further validated by *in vivo* comparison of embryonic tissues. For more accurate lineage assignments responsible for decisions of connected lineages sharing barcodes, we re-clustered cells within each lineage and then annotated “unclassified” cells per each lineage by less correlation. Low correlated cells were not used for constructing linkage maps.

#### Random Forest classification

For consistency of lineage annotation across different experiments, we calculated lineage prediction scores by applying Random Forest classifiers based on 10 lineages of timed EB data as in previous works (van Galen et al., 2019). In brief, we trained 1,000 trees over timed EB data using all expressed genes to classify all 10 lineages as in Figures 1 and 2. We then further trained the 1,000 trees by selecting the most informative genes (n = 1000) and validated them by performing 5 random subsets (5.52% error rate). We applied this classifier to our perturbation data in Figure 6. For the parallel sequencing data in Figure 5, we selected 5 lineages from timed EB data at day 14 and trained Random Forest classifiers of late EBs (day 10–14) as analyzed above (6.53% error rate). To calculate lineage prediction scores, we applied a defined Random Forest classifiers to our parallel sequencing and 5-azacytidine perturbation datasets and displayed prediction scores (ranging from 0 to 1) over previously clustered cells as a heatmap. We found weak prediction scores in blood progenitors (BP) and surface ectoderm (S.ECT) cells from the parallel sequencing dataset and in most clusters of 5-azacytidine perturbation dataset. Trained classifiers have limits to categorize new cell types if it is not included in the input lineages (pre-defined in timed EBs). Indeed, the Louvain clustering identified that BP was slightly further differentiated into Erythroid and Myeloid progenitor-like cells, and S.ECT was progressed with MES signature in deep transcriptomic profiling of 4224 cells. In 5-azacytidine perturbation dataset, there was a cluster that resemble the 2-cell embryonic stage and intermediates between mESC and PGC-like cells.

To map the 5-azacytidine perturbation dataset to the previously reconstructed EB trajectory, we used lineage prediction scores to identify the 10 most highly correlated cells of timed EB data. We then counted the frequency of identified cells and displayed its density on the trajectory.

#### Comparison of the *in vivo* and *in vitro* RNA-seq data

To compare the cellular populations inferred from scRNA-seq during EB differentiation to their *in vivo* counterpart, we downloaded bulk or single cell RNA-seq data corresponding to each embryonic layer from mouse embryos throughout development, and computed a correlation score for all populations that were common across our study and the dataset origin (Boroviak et al., 2015; Hargan-Calvopina et al., 2016; Nestorowa et al., 2016; Zhang et al., 2018). For bulk RNA-seq data of post-implantation tissue and PGC, we downloaded gene expression matrix with FPKM value and calculated average expression of replicates of the sample. For single cell RNA-seq data of HSC, we downloaded gene expression matrix with reads count and calculated TPM by normalizing to total read counts of each single cell. The average mRNA expression for all single cells belonging to one cell type is calculated. For single cell RNA-seq data of pre-implantation tissue, we downloaded gene expression matrix with FPKM and calculated average expression of all the single cell for one cell type is calculated. To compare the similarity between the *in vitro* and *in vivo* data, scRNA-seq data of EB were aggregated for each assigned lineage. The pair-wised Spearman’s correlation between aggregated single cell EB data and *in vivo* bulk data was calculated based on the lineage genes identified from clustering (P value < 1e-5).

#### Gene modules in branching points

To find gene expression modules that change along pseudotime and following its expression trends at each branch point, we applied branched expression analysis modeling using Monocle 2 (version 2.2.0). In brief, we extracted branch dependent genes with a significance score (q-value < 1e-4) over different branches at the branch point, and then aligned hierarchically clustered genes along pseudotime points from center (root) to both edges (bifurcated branches). We next identified clustered genes (gene modules) by the corresponding lineage marker genes we used in previous lineage annotation. We summarized expression trends of each module by regression analysis (LOESS) that created a smooth line through each expression score scatterplot. With this trend plot, we dissected gene modules to early and late activation clusters along pseudotime progression and revealed mutually exclusive clusters over branches.

#### DNA methylation data analysis

To identify the genes activated upon 5-azacytidine treatment, we compared the TPM of all the single cell profiles between 5-azacytidine treatment and DMSO from day2 to day14. The activated genes in 5-azacytidine treatment have 2-fold increase of their average expression across all the cells (P value < 1e-3). To examine the DNA methylation level on the promoters of genes activated upon 5aza treatment, we download the bulk WGBS data corresponding to the different lineages of the mouse embryo (Zhang et al., 2018). The average of mCG level on the 5kb of promoters were calculated and mapped to the activated genes.

## Supplementary Material

1

2

3

4

5

6

## Figures and Tables

**Table:** KEY RESOURCES TABLE

REAGENT or RESOURCE Antibodies	SOURCE	IDENTIFIER
Antibodies		
Anti-Mouse CD140a (PDGF Receptor a) APC	eBioscience	17–1401-81; RRID:AB_529482
BV421 Rat Anti-Mouse CD184(Cxcr4)	BD bioscience	562738; RRID:AB_2737757
Anti-Mouse CD133 (Prominin-1) PE	eBioscience	12–1331-80; RRID:AB_465848
Human/Mouse SSEA-1 Alexa Fluor 700 mAb (Cl MC-480)	R&D systems	FAB2155N-025 (No RRID number available)
Chemicals, Peptides, and Recombinant Proteins		
DMSO	Sigma	D5879
(Z)-4-Hydroxytamoxifen	Sigma	H7904
5azacytidine	Sigma	A2385–100MG
AMPure XP (SPRI) beads	Beckman Coulter	A63881
Sodium hydroxide	Sigma	S8045–500G
UltraPure Distilled Water	ThermoFisher	10977015
Sodium Chloride, 5M	Broad Institute	N/A
EDTA (0.5M, pH 8.0)	Broad Institute	N/A
2-Mercaptoethanol	Sigma	6010
BSA	Sigma	A9418
PEG 20%, Sodium Chloride 2.5M(L)	Broad Institute	N/A
Calcium/Magnesium free PBS	ThermoFisher	10010023
ESGRO® Leukemia Inhibitory Factor (LIF)	Millipore	ESG1107
HyClone Fetal Bovine Serum (U.S.), Embryonic Stem (ES) Cell Screened	GE healthcare	SH30070.02E
Tris-acetate	Broad Institute	N/A
Potassium Acetate	Broad Institute	N/A
Magnesium Acetate	Broad Institute	N/A
ExoSAP-IT	ThermoFisher	78200
E.coli DNA polymerase I	ThermoFisher	18010–025
RNaseH	ThermoFisher	18021–071
E.coli DNA ligase	ThermoFisher	18052–019
10mM dNTP mixture	ThermoFisher	R0192
Second strand buffer	ThermoFisher	10812–014
RNaseOUT	ThermoFisher	10777–019
IGEPAL	Sigma	I8896
Proteinase-K	ThermoFisher	EO0491
DNA Q5 Hot Star Hifi 500	New England BioLabs	M0493L
Critical Commercial Assays		
LIVE/DEAD® Fixable Aqua Dead Cell Stain Kit	ThermoFisher	L34957
LIVE/DEAD® Fixable Near-IR Dead Cell Stain Kit	ThermoFisher	L10119
PfuUltra II Hotstart PCR Master Mix	Agilent	600852
Qubit dsDNA HS Assay Kit	ThermoFisher	Q32854
BioA High Sensitivity DNA Kit	Agilent	5067–4626
Lipofectamine® 3000 Reagent	ThermoFisher	L3000008
NEBNext Ultra II End-Repair/dA-tailing Module	New England BioLabs	E7645
Blunt/TA Ligase Master Mix	New England BioLabs	M0367
Aggrewell 400	STEMCELL technologies	27945
MEGAscript T7 Transcription Kit	ThermoFisher	AM1334
High-Fidelity PCR Master Mix	New England BioLabs	M0531S
Superscripts	ThermoFisher	18064–014
1D Ligation Sequencing Kit	Oxford Nanopore Technologies	SQK-LSK109
1D Native barcoding kit	Oxford Nanopore Technologies	EXP-NBD104, EXP-NBD114
Deposited Data		
Raw data	GEO	GSE140890
Processed data	GEO	GSE140890
Experimental Models: Cell Lines			
mESC	Broad Institute	N/A
C57BL/6 MEF 4M IRR	GlobalStem	GSC-6002G
Oligonucleotides			
See Table S2 for a list of oligonucleotide sequences.		N/A
Recombinant DNA			
VSV.G	Broad institute	N/A
dVPR	Broad institute	N/A
p-EF1a-CreERT2–3Xflag-T2A-eBFP2	Table S1	N/A
p-EF1a-PuroR-T2A-E2Crimson-tandemLoxp-Filler	Table S1	N/A
p-EF1a-fl-mRFP-HSVpa-fl-MCS-T2A-eGFP	Table S1	N/A
Software and Algorithms			
R version 3.3	R Core Team	https://www.r-project.org
R package - randomForest	CRAN	https://cran.r-project.org/web/packages/randomForest/index.html
R package - circlize	CRAN	https://cran.r-project.org/web/packages/circlize/index.html
R package - Monocle version 2.2.0	CRAN	https://bioconductor.org/packages/release/bioc/html/monocle.html
Seurat version 2.2.1	Github	https://github.com/satijalab/seurat
FlowJo version 10.4.2	TreeStar	https://www.flowjo.com
Albacore version 2.3.3	Github	https://github.com/dvera/albacore
Guppy version 2.3.5	Oxford Nanopore Technology	https://community.nanoporetech.com/sso/login?next_url=%2Fdownloads
Minimap2	Github	https://github.com/lh3/minimap2
celseq2	Github	https://github.com/yanailab/celseq2

## Highlights

- Single-cell trajectory map of the embryoid body model of early embryogenesis
- A temporally precise genetic recording system for lineage tracing
- PGC-like lineage commitment in EBs occurs at the preimplantation epiblast-like stage
- DNA methylation determines PGC-fate choice in a narrow developmental window
