# Supplementary material for: Parallel Single-Cell RNA-Seq and Genetic Recording Reveals Lineage Decisions in Developing Embryoid Bodies
Source: Cell Rep. Author manuscript; Available in PMC 2020 Nov 6. (PMC7646252; doi:10.1016/j.celrep.2020.108222)
Supplement: 1 [file NIHMS1639623-supplement-1.pdf]

Cell Reports, Volume *33*

Supplemental Information

Parallel Single-Cell RNA-Seq

and Genetic Recording Reveals Lineage

Decisions in Developing Embryoid Bodies

Ik Soo Kim, Jingyi Wu, Gilbert J. Rahme, Sofia Battaglia, Atray Dixit, Elizabeth Gaskell, Huidong Chen, Luca Pinello, and Bradley E. Bernstein

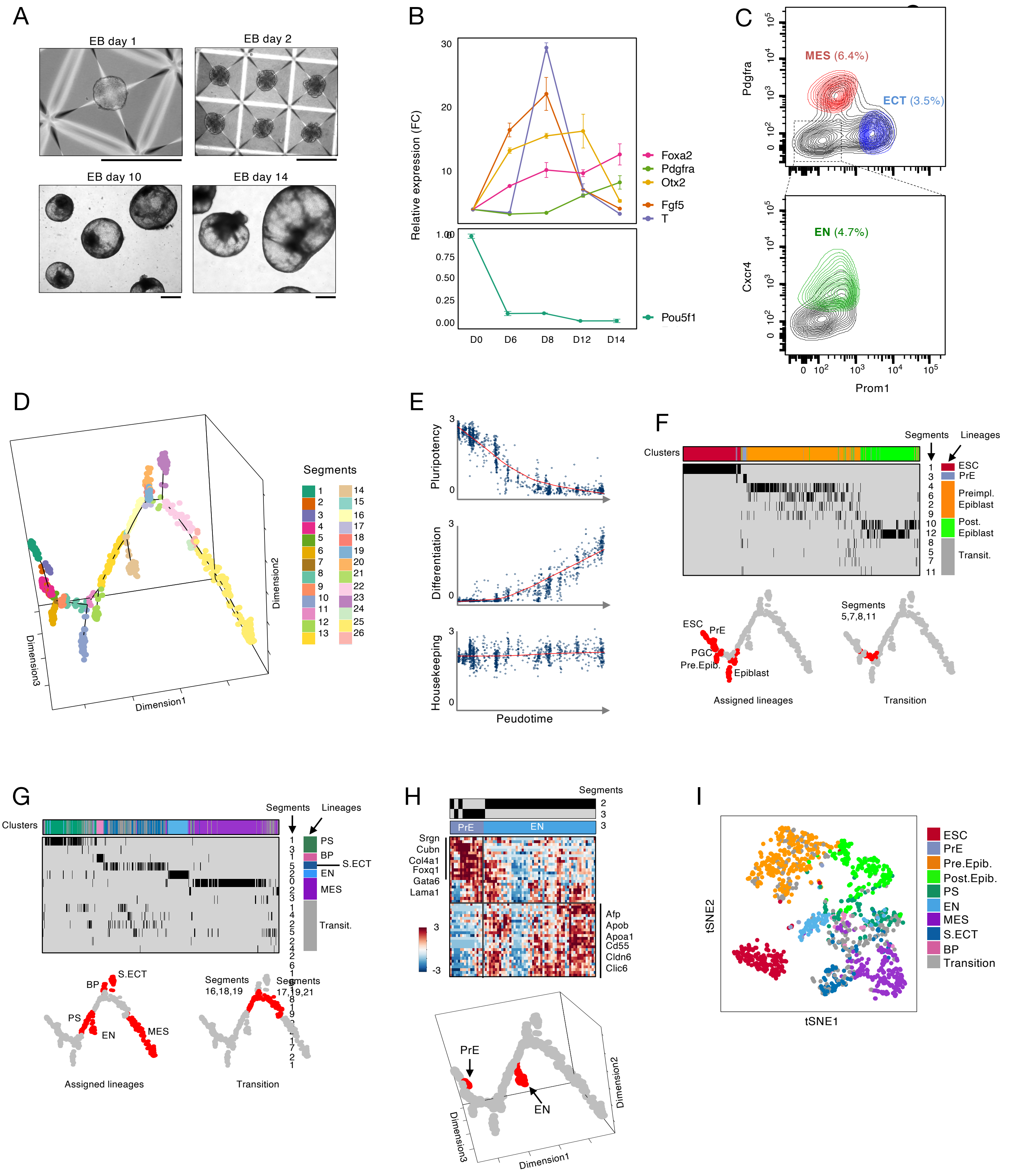
A
EB day 1
EB day 2
EB day 10
EB day 14
B
Relative expression (FC)
Foxa2
Pdgfra
Otx2
Fgf5
T
Pou5f1
D0
D6
D8
D12
D14
C
Pdgfra
MES (6.4%)
ECT (3.5%)
EN (4.7%)
Cxcr4
Prom1
D
Segments
Dimension1
Dimension2
Dimension3
E
Pluripotency
Differentiation
Housekeeping
Peudotime
F
Clusters
Segments
Lineages
ESC
PrE
Preimpl. Epiblast
Post. Epiblast
Transit.
PGC
Pre.Epib.
Epiblast
Segments 5,7,8,11
Assigned lineages
Transition
G
Clusters
Segments
Lineages
PS
BP
S.ECT
EN
MES
Transit.
Segments 16,18,19
Segments 17,19,21
Assigned lineages
Transition
H
Segments
PrE
EN
Srgn
Cubn
Col4a1
Foxq1
Gata6
Lama1
Afp
Apob
Apoa1
Cd55
Cldn6
Clic6
Dimension1
Dimension2
Dimension3
I
tSNE1
tSNE2
ESC
PrE
Pre.Epib.
Post.Epib.
PS
EN
MES
S.ECT
BP
Transition

Figure S1

Figure S1. EB construction and refined lineage annotation (Related to Figure 1)
(A) Representative images of EB progression over 14 days of spontaneous differentiation. EB days 1&2 are shown on the micro-well plate and EB days 10&14 are shown on the uncoated culture dish. Scale bar: 400um.
(B) Relative expression of marker genes of pluripotency and embryo development from RNA-seq for bulk EBs (10 EBs per time point). Gradual decrease of pluripotency genes (Pou5f1) and sequential activation of marker genes of developing embryo confirmed spontaneous EB differentiation into germ layers: Fgf5 (Post-Epib.), T (PS), Foxa2 (EN), Pdgfra (MES) and Otx2 (ECT).
(C) FACS analysis of EBs at day 14 stained by antibodies of germ layer marker genes: Pdgfra (Mesoderm), Cxcr4 (Endoderm), Prom1 (Ectoderm)
(D) Monocle predicts a trajectory consisting of 26 segments.
(E) Trend plots depicting the average expression of pluripotency markers, differentiation markers, and housekeeping genes plotted as a function of pseudotime. Pluripotency: Pou5f1, Nanog, Dppa5a, Utf1, Fgf4, Dppa4; Differentiation: Hand1, Col1a2, Acta2, Hand2, Snai2, Twist2; Housekeeping: Gapdh, B2m, Tbp, Actb.
(F) Refined lineage assignment of early EBs (from ESC to Postimplantation Epiblast; Segments 1~12) according to distribution of segments over cell clusters (as in Fig.1D). A group of cells spread out several different clusters are marked as a transition (Segments 5,7,8,11).
(G) Refined lineage assignment of late EBs (from PS to MES; Segments 13~26) according to distribution of segments over cell clusters (as in Fig.1E). A group of cells spread out several different lineages are marked as a transition (Segments 15,17,21,22,24,26).
(H) Heatmap depicts unbiased hierarchical clustering of single cell transcriptomes from PrE and EN cells only. Differentially expressed genes (P-value < 1e-3) are represented on the side of a heatmap.
(I) t-distributed stochastic neighbor embedding (tSNE) plot with refined lineage assignment from Fig 1. Cells are color coded based on the clustering in Fig.1B.

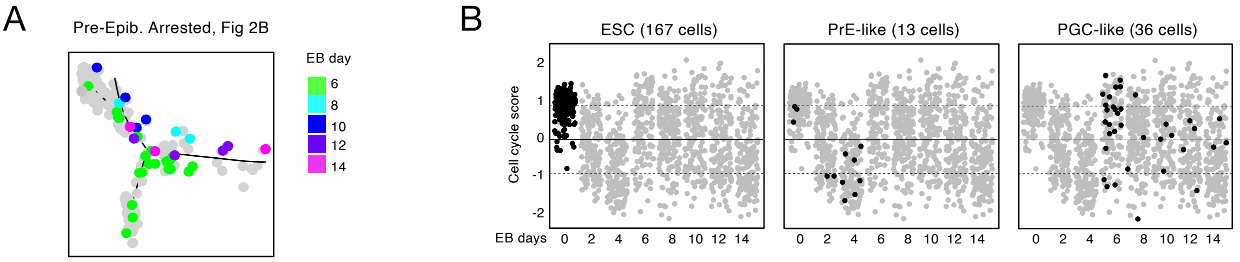

C

GO	Terms	PValue	Genes
GO:0007140	Male meiosis	0.002	Tex14,Tex15,Tdrd12
GO:0008152	Metabolic process	0.005	Alpl,Gstm3,Enpp3,Gstp2,Alg13,Gstp1
GO:0009566	Fertilization	0.006	Tex15,Ooep,Tdrd12
GO:0007275	Multicellular organism development	0.011	Elf3,Nphs1,Prdm1,Tdrd12,Dppa3,C77370,Morc1,Dppa4
GO:0019827	Stem cell population maintenance	0.013	Rif1,Mtf2,Tfap2c
GO:2000653	Regulation of genetic imprinting	0.017	Zfp42,Dppa3
GO:0051321	Meiotic cell cycle	0.022	Tex15,Tdrd12,Smc1b
GO:0001701	In Utero embryonic development	0.036	Ooep,Zfp42,Prdm1,Klf2
GO:0030154	Cell differentiation	0.040	Tex15,Elf3,Tfap2c,Tdrd12,Morc1,Setx
GO:0043046	DNA methylation involved in gamete generation	0.043	Tdrd12,Morc1
GO:0007283	Spermatogenesis	0.076	Tex15,Tdrd12,Morc1,Setx

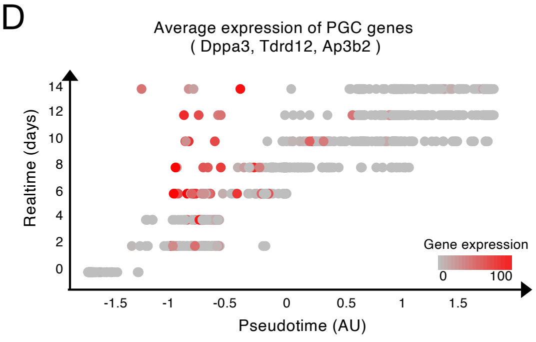

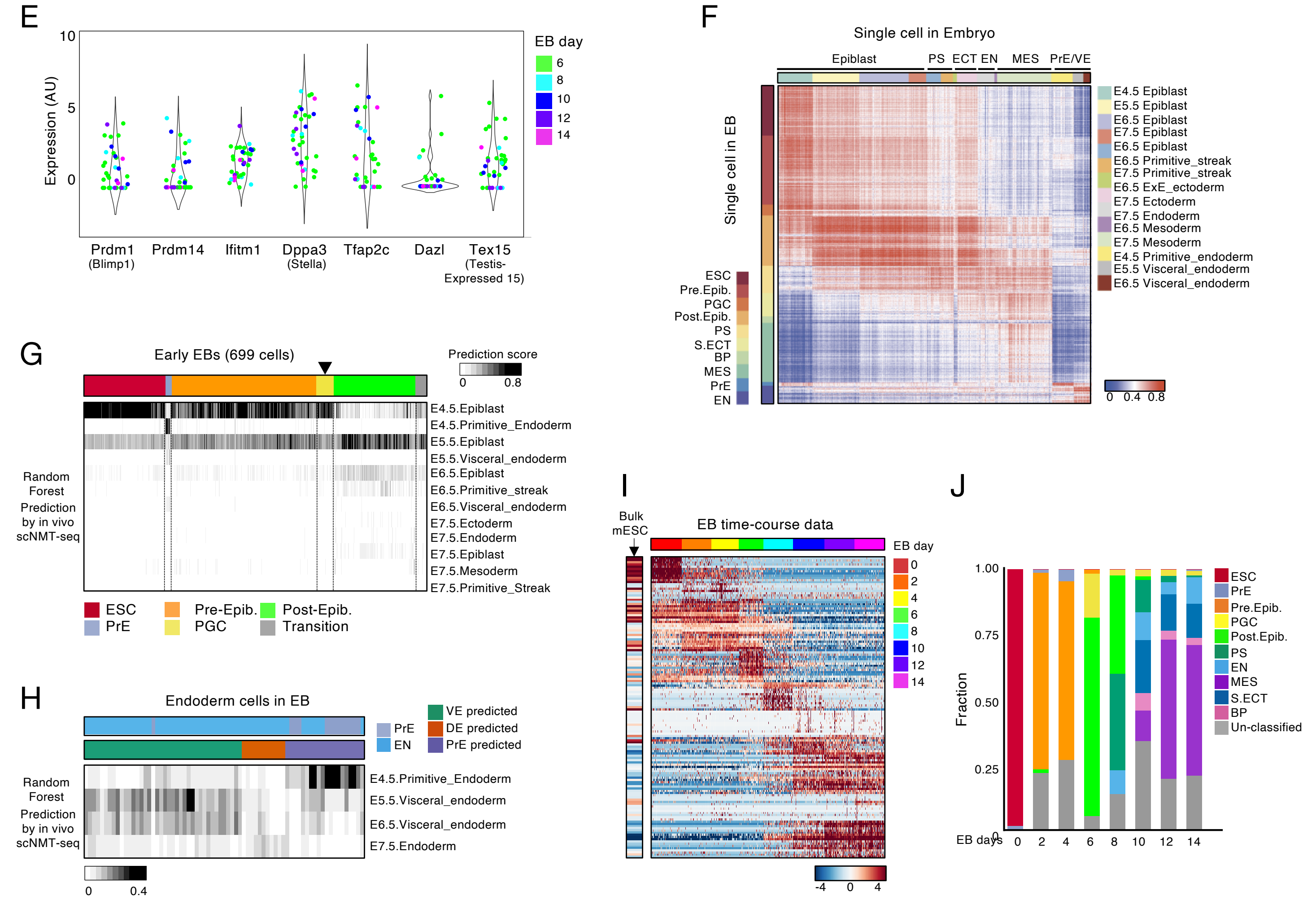

Figure S2

Figure S2. Annotation of "arrested" population as PGC-like cells and distributions of refined lineages in EB progression (Related to Figure 2)

(A) Map shows projection of arrested cells (colored circles) onto the zoomed trajectory plot of Fig 2B (gray circles). Arrested cells are colored by day of collection.

(B) Dot plots depict cell cycle score (Y-axis) for cells collected at successive days of EB differentiation (x-axis). Respective plots from left to right highlight ESCs, PrE and PGC-like cells in black. PGC-like cells exhibit proliferation signatures intermediate between fast (ESC) and arrested (PrE day 2/4) populations.

(C) Gene ontology terms of top 30 differentially expressed genes (P-value < 1e-5) from the population of "arrested" cells. Germ-cell related terms highlighted (yellow).

(D) Plot compares real-time point of collection (y-axis) against pseudo-time score (x-axis) for 1,536 single cells (points), as in Fig. 2A. Red heat indicates average expression of PGC-like cell marker genes (Dppa3, Tdrd12 and Ap3b2).

(E) Violin plot shows expression of known PGC marker genes in PGC-like cells isolated at indicated days in the EB time-course (color coded as in Fig.1B).

(F) Heatmap shows expression correlations between single cells from EBs (rows) and single cells from early embryos (columns) (Argelaguet et al., 2019). Correlations were computed over differentially-expressed genes that defined EB lineages (STAR methods).

(G) Heatmap shows lineage prediction scores (gray heat) for single cells in early EBs (columns). Prediction scores derived by random forest classifiers reflect the extent to which single cell transcriptomes correspond to the indicated lineage annotations (rows), based on single-cell RNA-seq for the early mouse embryo (Argelaguet et al. 2019).

(H) Heatmap shows lineage prediction scores (gray heat) for single cells from the EB dataset assigned as endoderm or primitive endoderm (columns). Prediction scores reflect the extent to which single cell transcriptomes correspond to the indicated endoderm lineage (rows), based on single-cell RNA-seq for mouse embryos (Argelaguet et al. 2019).

(I) Heatmap shows differentially expressed genes between timepoints across the EB differentiation time-course. Each column reflects a single cell, ordered by day of collection, and each row reflects a differentially-expressed gene. Narrow heatmap in the left shows the expression of these genes in bulk RNA-seq of mESC (Wu et al. 2016).

(J) Stacked bar plot shows fraction of single-cells in EBs assigned to indicated lineages. Data are stratified by day of collection (color coded as in Fig.1B).

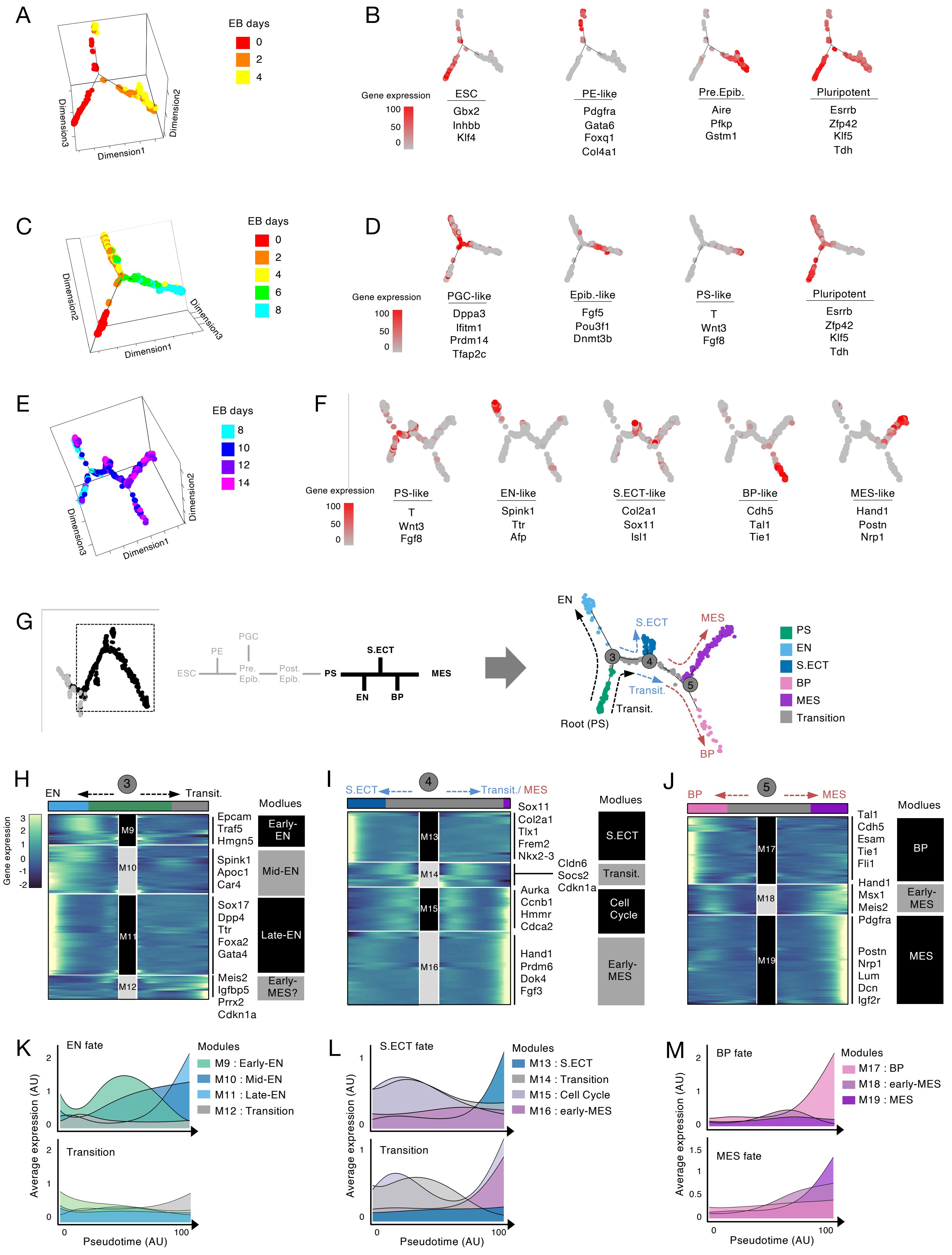
A
EB days
0
2
4
Dimension1
Dimension2
Dimension3
B
Gene expression
100
50
0
ESC
Gbx2
Inhbb
Klf4
PE-like
Pdgfra
Gata6
Foxq1
Col4a1
Pre.Epib.
Aire
Pfkp
Gstm1
Pluripotent
Esrrb
Zfp42
Klf5
Tdh
C
EB days
0
2
4
6
8
D
PGC-like
Dppa3
Ifitm1
Prdm14
Tfap2c
Epib.-like
Fgf5
Pou3f1
Dnmt3b
PS-like
T
Wnt3
Fgf8
Pluripotent
Esrrb
Zfp42
Klf5
Tdh
E
EB days
8
10
12
14
F
PS-like
T
Wnt3
Fgf8
EN-like
Spink1
Ttr
Afp
S.ECT-like
Col2a1
Sox11
Isl1
BP-like
Cdh5
Tal1
Tie1
MES-like
Hand1
Postn
Nrp1
G
PGC
PE
ESC
Pre.
Epib.
Post.
Epib.
PS
S.ECT
MES
EN
BP
EN
S.ECT
MES
Transit.
Transit.
Root (PS)
BP
PS
EN
S.ECT
BP
MES
Transition
H
EN
3
Transit.
Modlues
Gene expression
M9
M10
M11
M12
Epcam
Traf5
Hmgn5
Early-EN
Spink1
Apoc1
Car4
Mid-EN
Sox17
Dpp4
Ttr
Foxa2
Gata4
Late-EN
Meis2
Igfbp5
Prrx2
Cdkn1a
Early-MES?
I
S.ECT
4
Transit./ MES
Modlues
M13
M14
M15
M16
Sox11
Col2a1
Tlx1
Frem2
Nkx2-3
S.ECT
Cldn6
Socs2
Cdkn1a
Transit.
Aurka
Ccnb1
Hmmr
Cdca2
Cell Cycle
Hand1
Prdm6
Dok4
Fgf3
Early-MES
J
BP
5
MES
Modlues
M17
M18
M19
Tal1
Cdh5
Esam
Tie1
Fli1
BP
Hand1
Msx1
Meis2
Early-MES
Pdgfra
Postn
Nrp1
Lum
Dcn
Igf2r
MES
K
EN fate
Modules
M9 : Early-EN
M10 : Mid-EN
M11 : Late-EN
M12 : Transition
Transition
Average expression (AU)
Pseudotime (AU)
L
S.ECT fate
Modules
M13 : S.ECT
M14 : Transition
M15 : Cell Cycle
M16 : early-MES
Transition
M
BP fate
Modules
M17 : BP
M18 : early-MES
M19 : MES
MES fate

Figure S3

Figure S3. Transcription modules at major lineage branch points (Related to Figure 3)
(A) Pseudo-time trajectory from Fig1B first lineage bifurcation only - with cells from each real-time point superimposed in red (day 0), orange (day 2) and yellow (day 4).
(B) Expression intensity of representative differentially expressed genes from each cell state projected onto the trajectory from **A**.
(C) Pseudo-time trajectory from Fig1B second lineage bifurcation only - with cells from each real-time point superimposed in red (day 0), orange (day 2), yellow (day 4), green (day 6) and blue (day 8).
(D) Expression intensity of representative differentially expressed genes from each cell state projected onto the trajectory from **C**.
(E) Pseudo-time trajectory from Fig1B from day 8 onwards - with cells from each real-time point superimposed in light blue (day 8), dark blue (day 10), purple (day 12) and pink (day 14).
(F) Expression intensity of representative differentially expressed genes from each cell state projected onto the trajectory from **E**.
(G) Schematic represents the later lineage bifurcations to form the germ layers. Pseudo-time trajectory was replotted for the 800 single cell transcriptomes from day 8-14.
(H, I, J) Heatmaps show unbiased clustering of transcriptional programs for the single cell transcriptomes at each of the 3 branch points indicated. Cells are ordered by their pseudotime score radiating away from the progenitor population in the center. The top differentially expressed genes for each cluster are annotated (P-value < 1e-5).
(K, L, M) Graphs show the average expression of each gene expression module from each of H, I and J above.

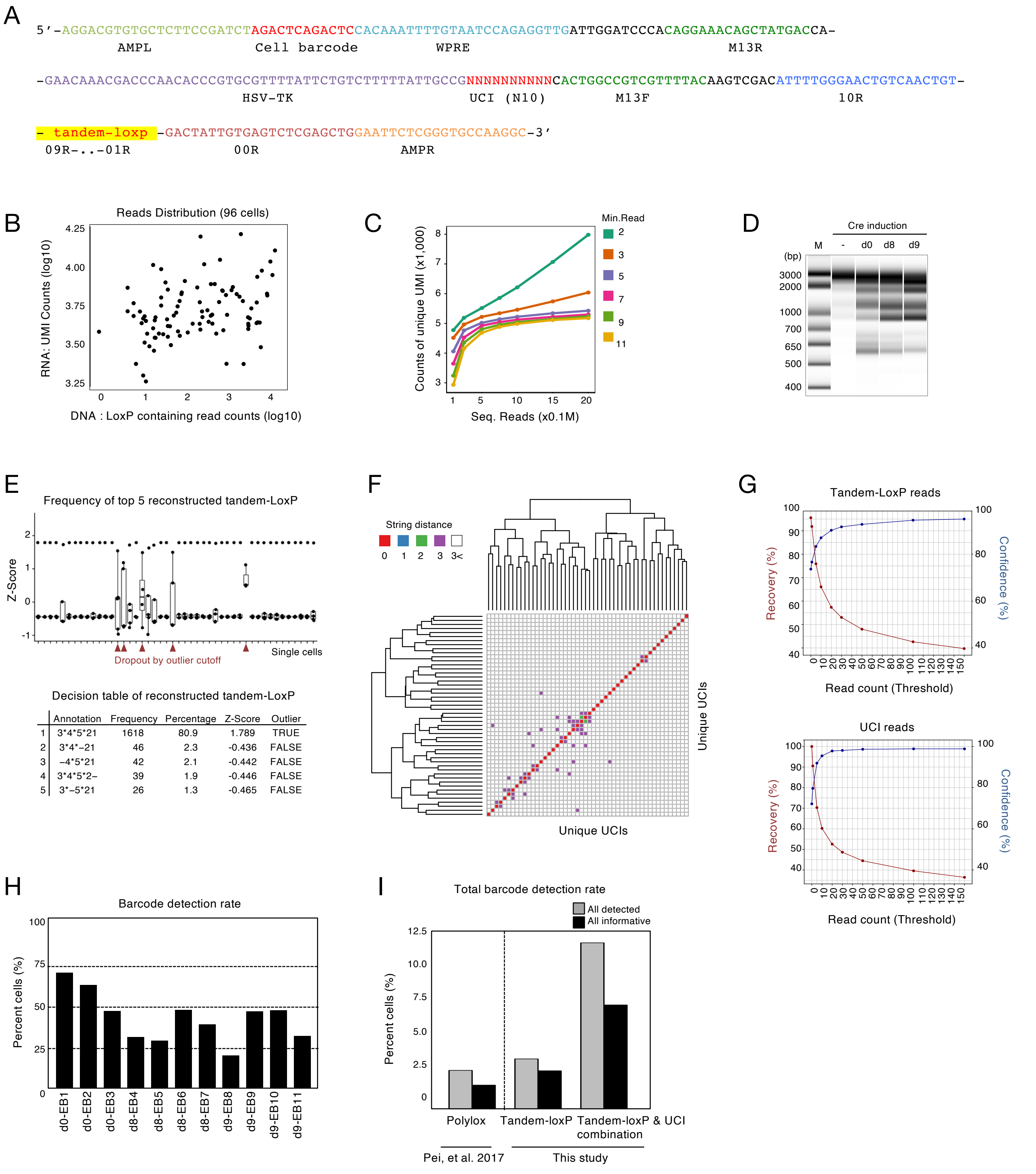

Decision table of reconstructed tandem-LoxP

	Annotation	Frequency	Percentage	Z-Score	Outlier
1	3*4*5*21	1618	80.9	1.789	TRUE
2	3*4*–21	46	2.3	-0.436	FALSE
3	–4*5*21	42	2.1	-0.442	FALSE
4	3*4*5*2–	39	1.9	-0.446	FALSE
5	3*–5*21	26	1.3	-0.465	FALSE

Figure S4

Figure S4. Dual-barcode extraction (Related to Figure 4)
(A) The sequence of amplified genetic barcode region containing both static and inducible barcodes.
(B) Scatter plot depicts the distribution of read counts from transcriptomes (short-read sequencing) and genetic barcodes (long-read sequencing) per single cell for 96 cells.
(C) Plot shows the predicted unique UCIs in virus particles. UCI reads are down sampled to 0.1 x 1e6~2 x 1e6 from 1e7 sequencing output and then counted by unique reads in different cutoffs of minimum-count.
(D) Gel shows the recombination efficiency after Cre induction at different time points.
(E) Reconstructed tandem LoxP sequences passing our strict QC criteria. The most frequent sequence per cell was accepted as a reconstructed tandem-LoxP only if it is classified as an outlier from the remaining "noise" sequencing data.
(F) Distance matrix of unique UCI sequences in EBs when barcodes are generated at day 8. All UCI sequences have at least 2 base differences.
(G) Threshold determination for the most confident and accurate tandem-loxp and UCI sequences per cell. Recovery rate is the percentage of cells after readcount cutoff. Confidence represents percent cells of reliable barcode over noise by outlier calculation.
(H) Barplot shows percent of cells in individual EBs for which detected barcodes could be assigned. Data are stratified by day of collection.
(I) Barplot shows percent of cells for which detected or informative barcodes could be assigned. Data are shown for the original report of LoxP-mediated barcoding system study (Pei et al 2017) and this study. Data reflect the percent of cells with informative barcodes (detected and retained after filtering out high frequency barcodes).

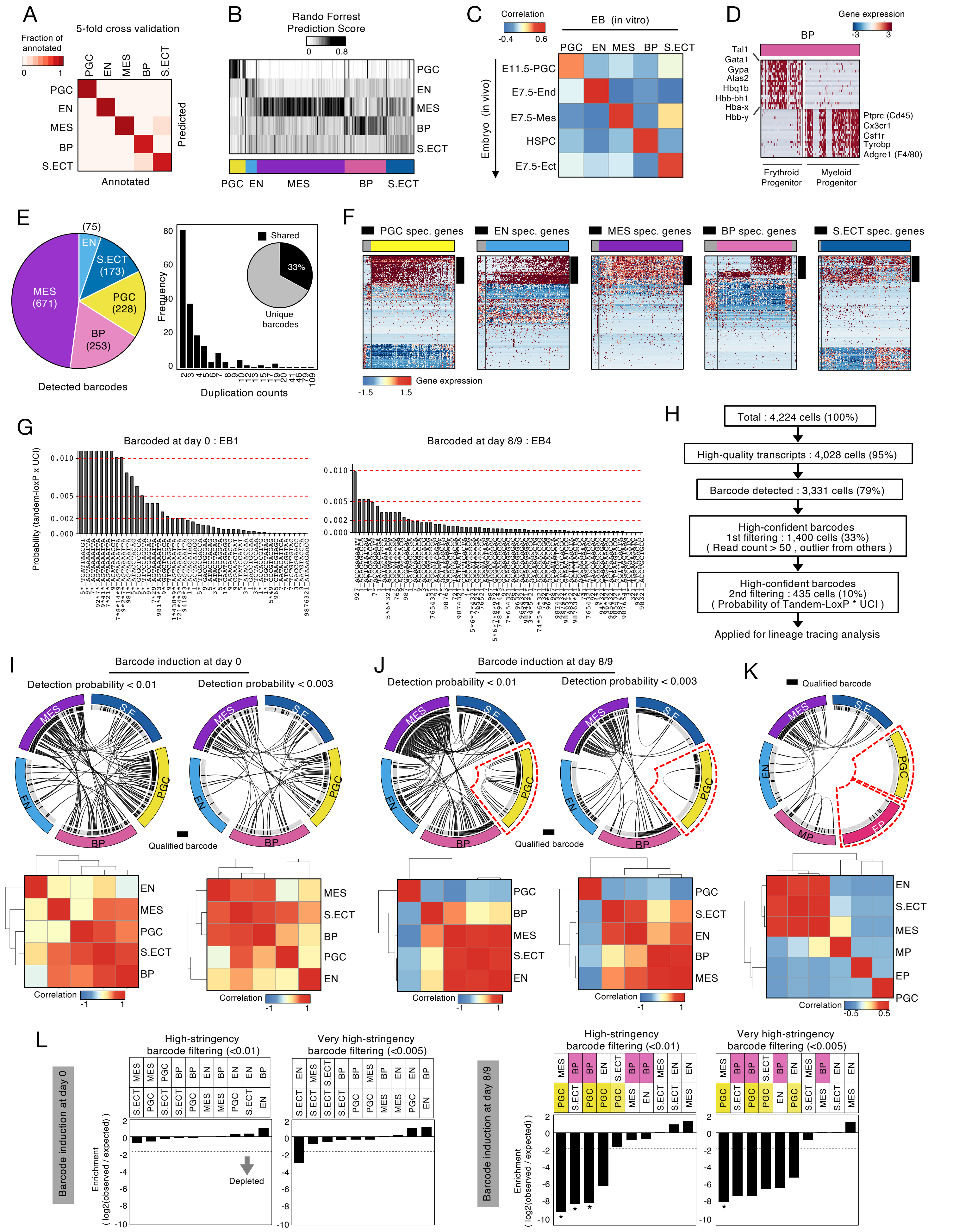
A
5-fold cross validation
Fraction of annotated
0
1
PGC
EN
MES
BP
S.ECT
Predicted
Annotated
B
Rando Forrest Prediction Score
0
0.8
PGC
EN
MES
BP
S.ECT
C
Correlation
-0.4
0.6
EB (in vitro)
Embryo (in vivo)
E11.5-PGC
E7.5-End
E7.5-Mes
HSPC
E7.5-Ect
D
Gene expression
-3
3
Tal1
Gata1
Gypa
Alas2
Hbq1b
Hbb-bh1
Hba-x
Hbb-y
Ptprc (Cd45)
Cx3cr1
Csf1r
Tyrobp
Adgre1 (F4/80)
Erythroid Progenitor
Myeloid Progenitor
E
(75)
EN
S.ECT (173)
MES (671)
PGC (228)
BP (253)
Detected barcodes
Frequency
Shared
33%
Unique barcodes
Duplication counts
F
PGC spec. genes
EN spec. genes
MES spec. genes
BP spec. genes
S.ECT spec. genes
Gene expression
-1.5
1.5
G
Barcoded at day 0 : EB1
Barcoded at day 8/9 : EB4
Probability (tandem-loxP x UCI)
H
Total : 4,224 cells (100%)
High-quality transcripts : 4,028 cells (95%)
Barcode detected : 3,331 cells (79%)
High-confident barcodes 1st filtering : 1,400 cells (33%) (Read count > 50 , outlier from others)
High-confident barcodes 2nd filtering : 435 cells (10%) (Probability of Tandem-LoxP * UCI)
Applied for lineage tracing analysis
I
Barcode induction at day 0
Detection probability < 0.01
Detection probability < 0.003
Qualified barcode
J
Barcode induction at day 8/9
Detection probability < 0.01
Detection probability < 0.003
Qualified barcode
K
Qualified barcode
MP
EP
Correlation
L
High-stringency barcode filtering (<0.01)
Very high-stringency barcode filtering (<0.005)
Barcode induction at day 0
Barcode induction at day 8/9
Enrichment (log2(observed / expected))
Depleted

Figure S5

Figure S5. Lineage assignment for barcoded EBs and lineage relationships from genetic barcoding data (Related to Figure 5)

(A) Heatmap displays results of a 5-fold cross-validation of the random forest classifier comprising major 5 classes corresponding to the cell types identified in the time-course EB data.

(B) Heatmap displays lineage annotation using random forest prediction scores for cells ordered as in Fig.5B.

(C) Correlation analysis of the annotated lineages with gene expression data from bulk *in vivo* cell lineages.

(D) Heatmap indicates Erythroid and Myeloid progenitor-like cells in BP-like cells. BP-like cells were further clustered to 2 sub-populations and each was annotated by known marker genes of Erythroid (Gata1, Gypa, Alas2 and Hbq1b) (Dzierzak and Bigas, 2018; Stadhouders et al., 2015) and Myeloid (Ptprc, Cx3cr1, Csf1r and Adgre1) (Bertrand et al., 2005))

(E) Pie-chart shows the number of total timestamp barcodes in each lineage before removing highly represented barcodes. Frequency of highly represented barcodes (shared) are counted (right).

(F) Heatmaps show re-clustering of each lineage based on top10 of all lineage marker genes. Cells that did not cluster well (Grey) with the majority were excluded for confident lineage assignment.

(G) Barcode probability of representative EBs. We calculated a barcode probability in each single EB separately by multiplying the probability of obtaining each observed genetic recorder sequence (tandem-loxP barcodes and UCI barcodes). We removed cells if the probability score was more than 0.005 (Star methods).

(H) Flowchart of barcode filtering shows the proportions of barcodes retained after each step.

(I-J) Linkage plots at high and low probability cutoff (0.01 and 0.003) showing robustness of lineage connections across probability cutoffs. PGCs showed a robust isolation from other cellular lineages across multiple probability cutoffs.

(K) Linkage plot including the EP and MP lineages. PGCs showed a robust isolation from other cellular lineages across multiple probability cutoffs.

(L) Barplot shows observed-to-expected enrichment analysis for each lineage pairs under different barcode filtering criteria (high-strigency:0.01; very high-strigency:0.005). Enrichment is calculated as log2 fold change between observed number of shared barcodes and expected number of shared barcodes. Significantly depleted pairs identified by permutation test (p-value < 0.03) are marked as asterisks in the plot (No lineage pairs are significantly enriched). Depletion indicates 75% reduction (enrichment = -2). PGC and BP lineage pairs are highlighted for d8/d9 treated samples.

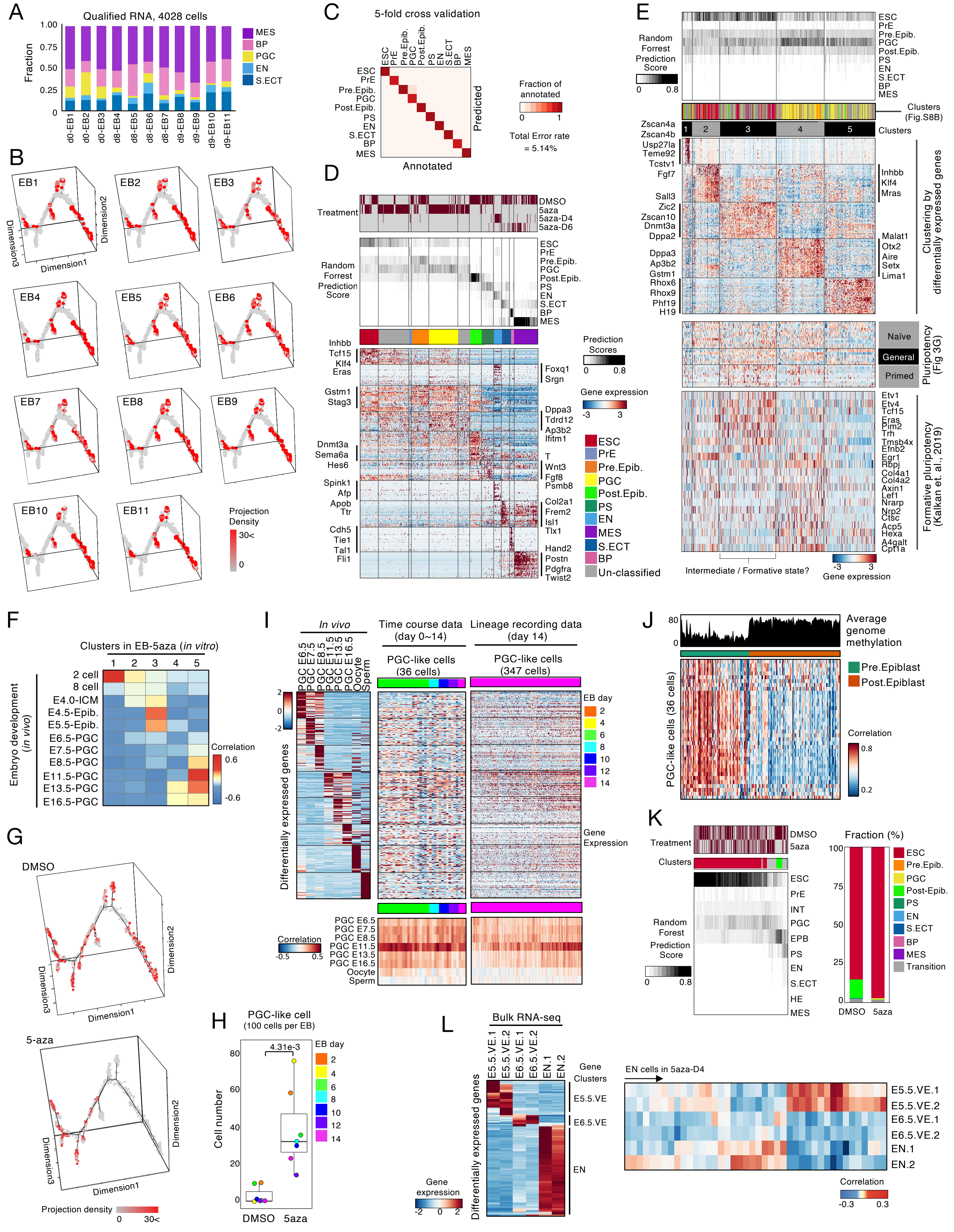
A
Qualified RNA, 4028 cells
Fraction
MES
BP
PGC
EN
S.ECT
B
Projection Density
C
5-fold cross validation
Annotated
Predicted
Fraction of annotated
Total Error rate = 5.14%
D
Treatment
DMSO
5aza
5aza-D4
5aza-D6
Random Forrest Prediction Score
Prediction Scores
Gene expression
Un-classified
E
Random Forrest Prediction Score
Clusters (Fig.S8B)
Clusters
Clustering by differentially expressed genes
Naïve
General
Primed
Pluripotency (Fig 3G)
Formative pluripotency (Kalkan et. al., 2019)
Intermediate / Formative state?
Gene expression
F
Clusters in EB-5aza (in vitro)
Embryo development (in vivo)
Correlation
G
DMSO
5-aza
Dimension1
Dimension2
Dimension3
Projection density
H
PGC-like cell (100 cells per EB)
4.31e-3
Cell number
EB day
DMSO
5aza
I
In vivo
Time course data (day 0~14)
Lineage recording data (day 14)
PGC-like cells (36 cells)
PGC-like cells (347 cells)
Differentially expressed genes
EB day
Gene Expression
Correlation
J
Average genome methylation
Pre.Epiblast
Post.Epiblast
PGC-like cells (36 cells)
Correlation
K
Treatment
Clusters
Random Forest Prediction Score
Fraction (%)
Transition
L
Bulk RNA-seq
Gene Clusters
Differentially expressed genes
Gene expression
EN cells in 5aza-D4
Correlation

Figure S6

Figure S6. DNA methylation dependent PGC-like cell fate decision and *in vivo* correlation of EB lineages : PGC and EN (Related to Figure 6)

(A) Stacked barplot shows fraction of single-cells in EBs assigned to indicated lineages. Data are stratified by EB and day of collection (color coded as in Fig.5B). All major lineages are present in each EB with comparable distribution.
(B) Individual cells per EB of timestamp data are projected on EB trajectory plot as shown in **Fig.1B.** Projection density indicates a frequency of highly correlated cells.
(C) Heatmap displays results of a 5-fold cross-validation of the random forest classifier comprising 10 classes corresponding to the cell types identified in the time-course EB data.
(D) Clustering and lineage annotation of DMSO or 5-azacytidine (5-aza) treated cells based on the top 10 differentially expressed genes. Top heatmap indicates the experimental condition. Middle heatmap depicts lineage annotation using random forest prediction scores. Bottom heatmap display top differentially expressed genes as in Fig.1 and 2 (P-value < 1e-3).
(E) Clustering and lineage annotation of cells from 5-aza-treated EBs using the top 10 differentially expressed genes. The 5 clusters represented are ESC (2), PGC-like cells(4,5) and mixed intermediates(3). Random Forest lineage prediction scores (top heatmap) indicate cell identities of cells transitioning between ESCs and PGCs. Bottom heatmaps display the expression of pluripotency genes of Fig 3G (top) and putative genes of pluripotency transition (bottom) (Formative phase; Kalkan et.al., 2019).
(F) Heatmap shows a correlation analysis of the clusters from 5-aza treated cells with gene expression data from bulk RNA-seq from isolated populations *in vivo* (Zhang et al., 2018; Wu et al., 2016; Magnúsdóttir et al., 2013; Seisenberger et al., 2012)
(G) DMSO or 5-aza treated cells are projected on the EB trajectory plot as shown in **Fig.1B**.
(H) t-test compare the number of PGCs in DMSO versus 5-aza treated EBs.
(I) Far left: Heatmap shows the expression of differentially expressed genes (DEGs) identified from PGC at E6.5 to E16.5 (Magnúsdóttir et al., 2013; Seisenberger et al., 2012) as well as mature germ cells (Hammoud et al., 2014; Wu et al., 2016). Right 2 panels: Heatmaps show the expression of the same DEGs for each single cell from PGC-like cells in EB data (left-time-course data, right-lineage tracing data). The bottom heatmap shows expression correlation scores between PGC-like cells and *in vivo* PGC/germ cells, based on the same DEGs.
(J) Heatmap shows expression correlation scores between PGC-like cells (rows) and *in vivo* epiblast cells at E4.5-5.5 (column) (Argelaguet et al., 2019). Correlations were computed using differentially-expressed genes that defined pre- or post-epiblast (STAR methods). Global methylation of each *in vivo* single cell is labeled above the heatmap.
(K) Left heatmap shows the expression of DEGs identified from VE and DE *in vivo (Zhang et al., 2018).* Right heatmap shows expression correlation scores between single cells from 5aza-D4 data assigned as endoderm (column) and *in vivo* VE and DE (row), based on the same DEGs.
(L) Heatmap shows lineage prediction scores (gray heat) for single cells in ESC treated by DMSO or 5-aza for 2 days (columns). Prediction scores reflect the extent to which single cell transcriptomes correspond to the indicated lineage annotations (rows), based on EB time-course data. Stacked bar plot shows fraction of single-cells in ESCs assigned to indicated lineages.
